# Insights Into *Limnothrix* sp. Metabolism Based on Comparative Genomics

**DOI:** 10.3389/fmicb.2018.02811

**Published:** 2018-11-20

**Authors:** Alex Ranieri Jerônimo Lima, Andrei Santos Siqueira, Janaina Mota de Vasconcelos, James Siqueira Pereira, Juliana Simão Nina de Azevedo, Pablo Henrique Gonçalves Moraes, Délia Cristina Figueira Aguiar, Clayton Pereira Silva de Lima, João Lídio Silva Gonçalves Vianez-Júnior, Márcio Roberto Teixeira Nunes, Luciana Pereira Xavier, Leonardo Teixeira Dall’Agnol, Evonnildo Costa Goncalves

**Affiliations:** ^1^Laboratório de Tecnologia Biomolecular, Instituto de Ciências Biológicas, Universidade Federal do Pará, Belém, Brazil; ^2^Laboratório de Biodiversidade Molecular, Universidade Federal Rural da Amazônia, Campus de Capanema, Capanema, Brazil; ^3^Centro de Inovações Tecnológicas, Instituto Evandro Chagas, Ananindeua, Brazil; ^4^Laboratório de Biotecnologia de Enzimas e Biotransformações, Instituto de Ciências Biológicas, Universidade Federal do Pará, Belém, Brazil; ^5^Grupo de Pesquisa em Biodiversidade, Bioprospecção e Biotecnologia, Universidade Federal do Maranhão, São Luís, Brazil

**Keywords:** Amazonian, cyanobacteria, *Limnothrix*, comparative genomics, genome mining

## Abstract

Currently only four genome sequences for *Limnothrix* spp. are publicly available, and information on the genetic properties of cyanobacteria belonging to this genus is limited. In this study, we report the draft genome of *Limnothrix* sp. CACIAM 69d, isolated from the reservoir of a hydroelectric dam located in the Amazon ecosystem, from where cyanobacterial genomic data are still scarce. Comparative genomic analysis of *Limnothrix* revealed the presence of key enzymes in the cyanobacterial central carbon metabolism and how it is well equipped for environmental sulfur and nitrogen acquisition. Additionally, this work covered the analysis of *Limnothrix* CRISPR-Cas systems, pathways related to biosynthesis of secondary metabolites and assembly of extracellular polymeric substances and their exportation. A *trans*-AT PKS gene cluster was identified in two strains, possibly related to the novel toxin Limnothrixin biosynthesis. Overall, the draft genome of *Limnothrix* sp. CACIAM 69d adds new data to the small *Limnothrix* genome library and contributes to a growing representativeness of cyanobacterial genomes from the Amazon region. The comparative genomic analysis of *Limnothrix* made it possible to highlight unique genes for each strain and understand the overall features of their metabolism.

## Introduction

The Cyanobacteria phylum (domain Bacteria) is one of the oldest and most morphologically diverse on the planet. The emergence of oxygen in the atmosphere, around 2.22–2.45 billion years ago, is attributed to the ability of cyanobacteria to perform photosynthesis (Schirrmeister et al., 2011). Given their metabolic diversity, cyanobacteria have a ubiquitous distribution, inhabiting various environments including freshwater, oceans, and soils. They also can be found in association with other organisms and in extreme environments, such as deserts or Antarctica (Vis, 2005; Komárek et al., 2012; Rigonato et al., 2012). Taxonomic studies, based on morphological criteria, have organized cyanobacteria into five subsections: (I) Chroococcales, (II) Pleurocapsales, (III) Oscillatoriales, (IV) Nostocales, and (V) Stigonematales (Rippka et al., 1979).

Cyanobacteria have been widely studied and exploited because of their biotechnological potential. Some cyanobacterial compounds have exhibited antibacterial, immunosuppressant, anticancer, anti-HIV and anti-fungal activities (Vijayakumar and Menakha, 2015). This ability to produce several bioactive compounds is mainly due to two biosynthetic enzyme complexes: NRPS (non-ribosomal peptide synthetases) and PKS (polyketide synthases). The NRPS complex can incorporate either proteinogenic or non-proteinogenic amino acids into the final peptide structure. Polyketides are biosynthesized by the PKS complex from acyl-CoA. Both enzyme complexes are similar in their biochemical logic, composing enzymes containing several modules, with each module performing a biochemical reaction in a sequential order. Thus, the order of modules determines the peptide or polyketide chain sequence (Micallef et al., 2015). One known exception is the *trans*-AT PKS system, which has no AT (acyltransferase) domain arranged in a modular fashion. Instead, the synthesized molecule receives an acyl building block through a free-standing AT (Helfrich and Piel, 2016). Additionally, the great diversity of genes encoding these enzymes can be combined to produce different metabolites (Nunnery et al., 2010).

Given their environmental and biotechnological importance, the number of available cyanobacterial genome sequences in GenBank has increased recently, from 340 reported genomes in 2015 (Land et al., 2015) to 838 as of April 2018 (GenBank prokaryotes.txt file). However, this number represents only 0.61% of the total bacterial genomes available. Despite recent attempts to increase the amount of available genomes in the five cyanobacteria subsections (Shih et al., 2013; Zhu et al., 2017), public genomic databases are lacking in geographical diversity, since many sequenced cyanobacteria are of European origin (Alvarenga et al., 2017).

**FIGURE 1:**
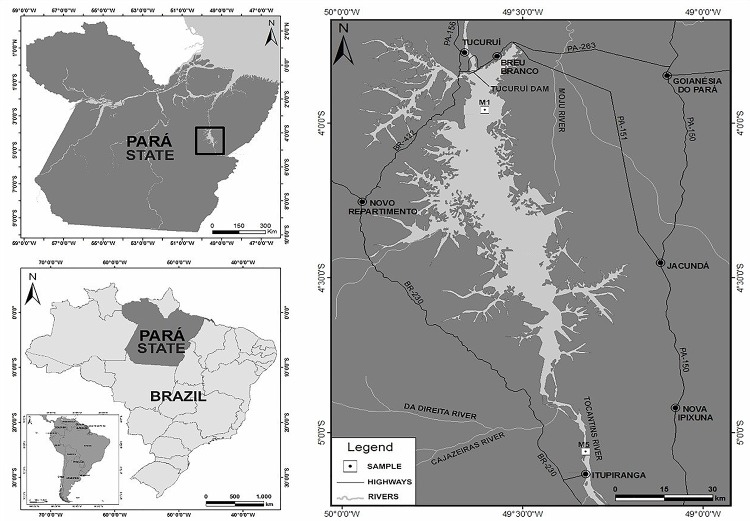
Map of Tucuruí Hydroelectric Dam, showing the M1 sampling point.

Although the Brazilian Amazon is known to harbor vast biodiversity, cyanobacterial genomic data are scarce for this biome (Rigonato et al., 2017). Previous metagenomic analysis conducted in Tucuruí Hydroelectric Reservoir (3°49′55″S, 49°38′50″W), which has an area of 1,783 km^2^ of flooded primary Amazonian forest (Fearnside, 2001) (Figure 1), revealed a high diversity of cyanobacteria (Baraúna et al., 2013; Das Graças et al., 2015). The reservoir has little human interference in its water quality (Curtarelli et al., 2016), and rainfall is the main source of nutrients when it is full, during the wet period from January to April; from March to December (the dry period) the nutrients are released from the lake bottom (Pettersson and Pozdnyakov, 2013). Due to rainfall changes, the pH and some nutrients such as nitrite, nitrate and phosphate may vary according to the seasons, affecting the average cyanobacterial abundance, which is higher in the wet period. Although the reservoir presents quite different physicochemical characteristics depending on sampling point, can be classified, in general, as mesotrophic with few oligotrophic sites (Brandão et al., 2017).

The high diversity of cyanobacteria, along with the distinct environmental characteristics of Tucuruí Hydroelectric Reservoir, led our group to isolate these microorganisms from that area, depositing them in the CACIAM collection (in Portuguese: *Coleção Amazônica de Cianobactérias e Microalgas* or Amazonian Collection of Cyanobacteria and Microalgae). Some representatives of the CACIAM collection have already been sequenced and published (Lima et al., 2014; Castro et al., 2016), including the genome of the newly described *Alkalinema* genus (Lima et al., 2017). Until the present study there were only three *Limnothrix* genomes available in public databases: *Limnothrix* sp. P13C2 (Tan et al., 2016), *Limnothrix* sp. PR1529 (GenBank: NZ_LIRO00000000) and *Limnothrix rosea* IAM M-220 NIES-208 (Zhu et al., 2017). In this work, we present the genome of *Limnothrix* sp. CACIAM 69d, isolated from Tucuruí Hydroelectric Reservoir (Figure 1).

The genus *Limnothrix* includes filamentous cyanobacteria, with no cellular differentiation or branching in their trichomes, presenting narrow cylindrical cells with polar and/or central aerotopes and very slight or non-existent motility (Zhu et al., 2012). This genus belongs to the cyanobacteria subsection III and has been classified within the order Oscillatoriales, being morphologically similar and phylogenetically closely related to *Pseudanabaena* spp. (Castenholz et al., 2015b). Some studies have been conducted with representatives of the genus *Limnothrix* in order to verify exopolysaccharides (EPS) (Khattar et al., 2010) and lipid production (Economou et al., 2015; Aboim et al., 2016; Hong et al., 2016; Oliveira et al., 2018), suggesting that this cyanobacteria could be used as a source for compounds of biotechnological interest. Besides that, *Limnothrix* (strain AC0243) is able to produce a toxin which causes severe toxicity to developing vertebrates with injuries particularly noted in the brain, notochord and pancreas, showing strong similarities to amino acid beta-*N*-methylamino-L-alanine (BMAA) intoxication (Daniels et al., 2014). Furthermore, another study revealed that the toxin produced by *Limnothrix* (strain AC0243) is capable of damaging the liver, lungs and gastrointestinal tract of mice. However, the authors were unable to isolate and identify this toxin (Humpage et al., 2012).

All these studies have been published in the absence of available genomes for *Limnothrix*, which may have limited the search for specific metabolites or their genes. Since the genomes are now available, it is possible to improve knowledge about this genus through genomic analysis. This approach is useful to gain insight into the diversity of cyanobacterial metabolism (Beck et al., 2012) and make it possible to identify several biosynthetic gene clusters which produce structurally diverse metabolites, such as alkaloids, terpenes, fatty acids, non-ribosomal peptides, UV-absorbing compounds, polyketides and ribosomal peptides (Micallef et al., 2015). Considering all those aspects, this work aims to perform a comparative analysis on the four available *Limnothrix* genomes, describing their metabolic characteristics and genome mining findings, emphasizing the strain CACIAM 69d.

## Materials and Methods

### *Limnothrix* Genome Data

*Limnothrix* genomes analyzed where obtained from GenBank, using the accession numbers: MBRF00000000 (*Limnothrix* sp. P13C2), NZ_LIRO00000000 (*Limnothrix* sp. PR1529) and NZ_MRBY00000000 (*Limnothrix rosea* IAM M-220 NIES-208).

The genome sequence of *Limnothrix* sp. CACIAM 69d has been deposited at DDBJ/ENA/GenBank under the accession MKGP00000000. The described in this paper is version MKGP00000000.2.

### Sample Collection and Non-axenic Cultivation of *Limnothrix* sp. CACIAM 69d

The isolate *Limnothrix* sp. strain CACIAM 69d was obtained from a water sample collected in December, 2010 from the M1 point of Tucuruí Hydroelectric Reservoir (3°49′55″S, 49°38′50″W), Pará, Brazil (Figure 1). Using a sterile plastic bottle, five liters of water were collected 50 cm below the lake surface. The water sample was sequentially filtered by negative pressure in four steps: prefiltration with qualitative filter paper grade 5 (Whatman, United Kingdom) and three consecutive filtrations using nitrocellulose membranes with pore diameters of 0.8 μm, 0.45 μm (Millipore, Brazil) and 0.2 μm (Whatman, United Kingdom); next, the 0.2 μm membrane containing the retained microorganisms was inoculated in BG-11 medium (Allen, 1968). After biomass had increased, the sample was inoculated in a cultivation medium containing cycloheximide (18.5 mg/mL) and then submitted to a five-step serial dilution process with factor 10. The sample was maintained in Erlenmeyer flasks at 29°C with a 13 h light/11 h dark regime. Monthly, an inoculum of *Limnothrix* sp. strain CACIAM 69d was transferred to another Erlenmeyer flask containing BG-11 medium.

### DNA Extraction, Genome Sequencing, Assembly, and Binning

The total DNA was extracted after 1 month of cultivation using a modified phenol–chloroform based protocol (Lin et al., 2010). One sequencing run was performed on a GS FLX 454 platform using a non-paired library, using standard protocols in 2011. The raw reads obtained were quality-filtered with a minimum Phred score of 20. Assembly was carried out using two tools: (i) Newbler v2.9 (Margulies et al., 2005), parameterized with minimum overlap of 40 bp, minimum overlap identity of 90%, heterozygote mode and extended low-depth overlap options; (ii) SPAdes 3.12.0 (Bankevich et al., 2012), using error correction and automatic k-mer estimation size. The assembly results were compared using QUAST (Gurevich et al., 2013).

Contigs from both assemblers were submitted to MaxBin 2.2 (Wu et al., 2015), MyCC (Lin and Liao, 2016), and MetaBAT 2 (Kang et al., 2015) for binning. CheckM (Parks et al., 2015) was used to obtain taxonomic classification of bins generated for each binning tool. Individually, for each assembly tool, bins identified as cyanobacteria were compared using checkM and QUAST to determine the best binning tool. Next, the best results of the best binning tool of each assembly were compared to choose the final representation of the assembly and binning process.

The cyanobacterial bin contamination assessment was performed by CheckM, identifying possible duplicated gene markers. BLASTp (Altschul et al., 1990) was used to identify which of the duplicated markers was a contamination. Next, genomic regions upstream and downstream of the contaminated marker gene were checked for contamination, using a preliminary annotation from RAST (Aziz et al., 2008) and BLASTp. Contaminated sequences were removed from the cyanobacterial bin and then a new contamination assess was performed by CheckM.

### Genome Annotation, Mining, and Comparative Analysis

For *Limnothrix* sp. CACIAM 69d genome, the annotation was performed by NCBI Prokaryotic Genome Annotation Pipeline (Tatusova et al., 2016). BLAST searches in BSRD database (Li et al., 2013) were executed to identify sRNA.

For all genomes, CRISPR arrays were identified by CRISPRDetect (Biswas et al., 2016), using default parameters. All unquestionable arrays considered by CRISPRDetect where selected for target prediction with CRISPRTarget (Biswas et al., 2013), comparing the spacer sequences against GenBank-Phage and RefSeq-Plasmid databases using default parameters. Whole genome BLAST search was performed against the COG database (Tatusov et al., 2003), using RPSBLAST with e-value of 1E-02. The genome mining process was carried out by a complete antiSMASH 4.0 (Blin et al., 2017) analysis, including whole-genome Pfam (Finn et al., 2014) searches. KS domain analysis was performed by NaPDoS (Ziemert et al., 2012), using the amino acid sequences of CDS presented in Table 4. Additional domain searches were performed by DELTA-BLAST against the NCBI’s Conserved Domain Database (CDD) (Marchler-Bauer et al., 2015). Metabolic pathway analysis was achieved by results obtained from a KEGG automatic annotation server KAAS (Moriya et al., 2007) and Reconstruct Pathway tool of KEGG Mapper. Additional searches where performed by BLAST using the amino acid sequences of *Limnothrix* sp. KNUA012 for AAR/ADO genes (GenBank accessions: KU341740 and KU341741) queries against translated CDS databases of each *Limnothrix* genome. For toxins, the following sequences where used as query (GenBank accession numbers): FJ477836.2 and JF803645.1 (anatoxin), KJ139742.1 (cylindrospermopsin), AY588942.1 (lyngbyatoxin), AY212249.1 and AF183408.1 (microcystin), AY210783.2 (nodularin), DQ787200.1 (saxitoxin). Pan-genome analysis was performed using the Bacterial Pan Genome Analysis Tool (BPGA) (Chaudhari et al., 2016), with default parameters.

### Phylogeny

**FIGURE 2:**
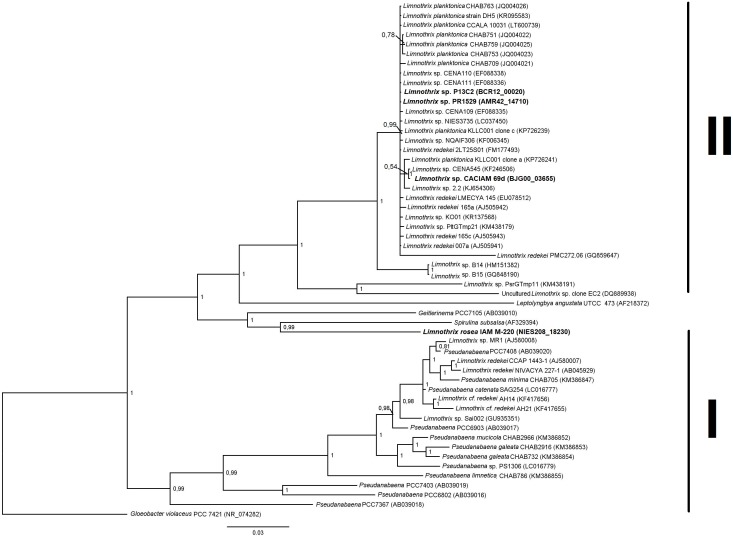
Bayesian phylogenetic tree based on 16S rRNA sequences from *Limnothrix* strains and other Oscillatorian genera. *Limnothrix* strains with sequenced genomes are shown in bold. *Gloeobacter violaceus* PCC 7421 was used as outgroup. GenBank accession numbers are in parentheses.

To generate the Bayesian tree, 16S rRNA sequences were obtained from NCBI and *Limnothrix* genomes. GenBank accession numbers for each sequence are shown in Figure 2. The sequences were aligned with an online version of MAFFT (Katoh et al., 2017). The nucleotide substitution model used was SYM+G, determined by PAUP 4.0b10 (Swofford, 2002) and MrModeltest 2 (Nylander, 2004). The tree was constructed using MrBayes 3.2.6 (Ronquist et al., 2012), running with 10^7^ generations, sampling every 100th iteration. TRACER 1.6 (Rambaut and Drummond, 2013) was used to check the performance of tree construction.

A phylogenetic tree of closely related genomes was constructed using the four *Limnothrix* genomes by the Species Tree App present in KBase. Briefly, this tool uses 49 highly conserved COG families to perform an alignment. The closest neighbors are extracted and concatenated to generate the tree using FastTree2, which is an approximation of the maximum likelihood method (Arkin et al., 2018).

## Results

### Genome Assembly and Binning

Results of CACIAM 69d assembly are shown in Supplementary Table 1. SPAdes assemblies were performed using 21, 33, 55, 77, 99, and 127 k-mer sizes due to the auto parameter. SPAdes k-127 assembly produced more contigs, a higher number of bases and the longest contig. Newbler 2.9 presented a larger N50 value, but also presented some mismatches (630 N’s) in the assembled contigs. Since the goal was to assemble the cyanobacterial genome, contigs from both Newbler 2.9 and SPAdes k-127 were selected to perform the binning process.

All three binning tools were able to recover a single cyanobacterial bin using contigs from both assemblers as input, showing the same percentage of genome completeness (99.64%) and contamination level (0.34%) (Bin01 in Table 1). Other bins are predominantly proteobacteria, presenting coverage values lower than 8x, completeness levels ranging from 6.8 to 99.73%, and contamination estimation from 0.11 to 45.53%.

**Table 1:** CheckM results for each assembler and binning tool.

Tool	MyCC		MaxBin 2.2		MetaBAT2	
			
Assembler	SPAdes	Newbler	SPAdes	Newbler	SPAdes	Newbler
Bin01	Cyanobacteria (99.64%, 0.34%, 22x)	Cyanobacteria (99.64%, 0.34%, 21x)	Cyanobacteria (99.64%, 0.34%, 23x)	Cyanobacteria (99.64%, 0.34%, 27x)	Cyanobacteria (99.64%, 0.34%, 22x)	Cyanobacteria (99.64%, 0.34%, 26x)
Bin02	Sphingomonadales (94.28%, 31.3%, 2.2x)	Sphingomonadales (98.44%, 21.1%, 2.7x)	Sphingomonadales (96.77%, 8.08%, 4x)	Sphingomonadales (98.34%, 5.13%, 4.6x)	Sphingomonadales (96.77%, 3.1%, 7.2%)	Sphingomonadales (98.27%, 0.97%, 8x)
Bin03	Bacteroidetes (91.98%, 4.3%, 4x)	Bacteroidetes (97.2%, 2.8%, 5.7x)	Bacteroidetes (95%, 16.35%, 3.6x)	Bacteroidetes (99.5%, 8.38%, 4.4x)	Bacteroidetes (92.65%. 0.99%, 6.2x)	Bacteroidetes (97.16%, 0.99%, 7x)
Bin04	Alphaproteobacteria (89.94%, 43.53%, 2x)	Alphaproteobacteria (92%, 7.81%, 4.2x)	–	Alphaproteobacteria (99.73%, 17.54%, 3.4x)	Alphaproteobacteria (98.91%, 0.65%, 6.7x)	Alphaproteobacteria (98.63%, 1.88%, 7.3x)
Bin05	Sphingomonadales (89.2%, 8.22%, 3.1x)	Sphingomonadales (92.68%, 18.29%, 3.3x)	Sphingomonadales (90.75%, 20.7%, 2.7x)	Sphingomonadales (91.53%, 23.25%, 3.2x)	Sphingomonadales (87.07%, 3.05%, 4.4x)	Sphingomonadales (87.08%, 2.02%, 4.8x)
Bin06	Burkholderiales (70.72%, 9.47%, 2.5x)	Burkholderiales (84.2%, 4.89%, 3x)	–	Burkholderiales (85.77%, 25.64%, 2.7x)	Burkholderiales (65.03%, 3.24%, 2.9x)	Burkholderiales (74.79%, 2.16%, 3.3x)
Bin07	Gammaproteobacteria (24.08%, 2.27%, 1.9x)	–	–	–	–	–
Bin08	Gammaproteobacteria (14.2%, 0.07%, 2x)	–	–	–	–	–
Bin09	Rhizobiales (15.61%, 1.85%, 2.1x)	Rhizobiales (9.08%, 0.72%, 2.4x)	–	–	–	–
Bin10	–	Xanthomonadaceae (11.3%, 0.08%, 2.3x)	–	–	Xanthomonadaceae (26.11%, 1.59%, 2.2x)	Xanthomonadaceae (25.4%, 0.11%, 2.5x)
Bin11	–	–	Sphingomonadales (7.31%, 1.05%, 2.1x)	–	–	–
Bin12	–	–	Pseudomonadales (7.31%, 1.05%, 2.1x)	–	–	–
Bin13	–	–	–	Burkholderiales (6.8%, 0.75%, 2.4x)	–	–


*The taxonomic rank is shown for each bin, including estimated completeness, contamination and coverage.*

After the comparative analyzes of the binning tools, MyCC revealed the best set of contigs for the cyanobacterial bins for both Newbler and SPAdes. This means that MyCC recovered a higher number of contigs and bases, besides containing sequences from other binning tools. Binning tool comparisons for Newbler contigs are available in Supplementary File 1; while the binning tool comparisons for SPAdes contigs are present in Supplementary File 2.

Metrics of the cyanobacterial bins recovered by MyCC from both Newbler and SPAdes contigs are shown in Supplementary Table 2. The bin from Newbler has more contigs and a higher number of bases, while the bin presented by SPAdes has the longest contig and a larger N50 value. An attempt to join both assemblies using Geneious 9.1.8 *de novo* Assemble tool (Kearse, 2012) revealed that 82 contigs from Newbler are contained in SPAdes contigs with more than 98% of similarity; 15 contigs from SPAdes are contained in Newbler contigs; and only 15 contigs overlapped, although with low similarity or adding only a few bases. In this way, the contigs assembled by SPAdes and binned by MyCC were chosen for further analysis.

Before the final version of the genome was obtained, checkM pointed out two duplicated marker genes suggesting a possible contamination. The marker PF09285.6 present at one end of contig MKGP02000001 was identified as a contamination and therefore removed, along with downstream and upstream regions not belonging to cyanobacteria. The other marker, TIGR01394, was found in the final portion of the second longest contig. After removing the contaminated region, the contig was split in MKGP02000002 and MKGP02000003. A new checkM analysis revealed no contamination present in the genome.

The draft genome of *Limnothrix* sp. strain CACIAM 69d has a total of 4.5 Mb distributed among 100 contigs, which range from 594 to 251,784 bp, with GC content of 55.23%, N50 of 86,917 bp and mean coverage of 22×. The NCBI prokaryotic genome annotation pipeline (Tatusova et al., 2016) identified 3,789 CDS, 42 tRNA, 2 rRNA, and 4 ncRNA. Additionally, an Yfr1 sRNA homolog with 64 bp was identified by BLAST searches.

### Phylogeny

The 16S rRNA gene present in *Limnothrix* sp. CACIAM 69d was identical to the one found in strain CENA545 and had 99.8% of identity with the 16S rRNA of *Limnothrix planctonica* KLLC001 clone a. Both *Limnothrix* sp. P13C2 and *Limnothrix* sp. PR1529 showed identical 16S rRNA sequences. These *Limnothrix* strains were grouped in the larger cluster II in a Bayesian tree (Figure 2). This cluster was formed mainly by strains from Asia, Europe, Africa, and America (Zhu et al., 2012).

**FIGURE 3:**
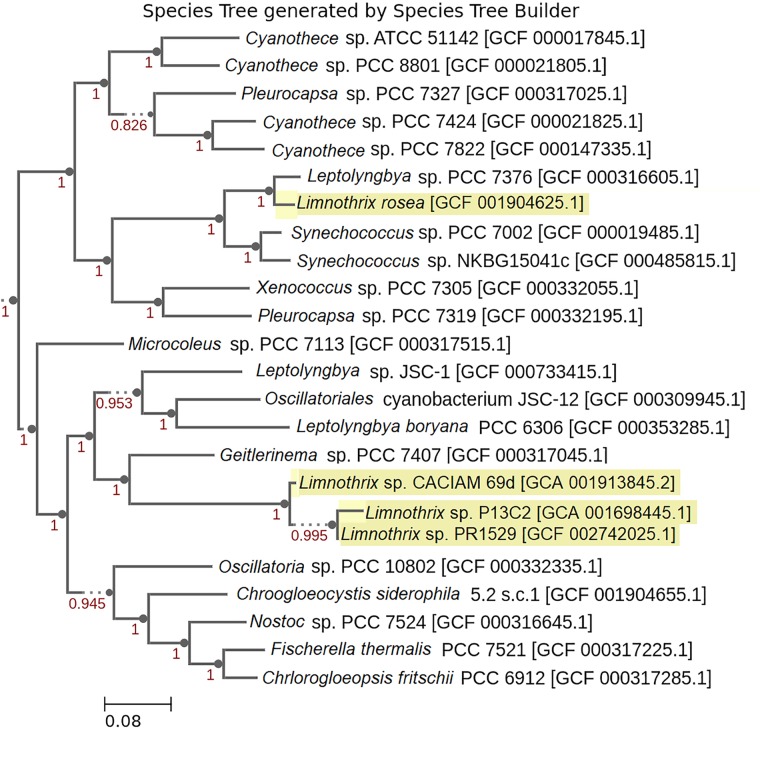
Phylogenetic Species Tree based on genomic sequences. *Limnothrix* strains are highlighted with a yellow background. The numbers presented are confidence values used by FastTree 2 (Price et al., 2010) to estimate maximum likelihood. GenBank and RefSeq assembly accessions are provided within the brackets.

Regarding *Limnothrix rosea* IAM M-220, it clustered together with *Spirulina subsalsa*, outside of the two formed clusters (Figure 2). A BLASTn search of *Limnothrix rosea* IAM M-220 16S rRNA sequence against the NCBI non-redundant nucleotide database revealed a 99% similarity with *Leptolyngbya* sp. PCC 7376, and also 98% of similarity with some *Synechococcus* strains, such as PCC 7117, PCC 8807 and PCC 73109. In this sense, *Limnothrix rosea* IAM M-220 was shown to be more similar to *Leptolyngbya* sp. PCC 7376 than other *Limnothrix* strains (Figure 3). The Species Tree reflects the same results obtained from BLASTn using the *Limnothrix rosea* IAM M-220 16S rRNA sequence.

### CRISPR-Cas Systems

The four *Limnothrix* genomes were scanned for Clustered Regularly Interspaced Short Palindromic Repeats (CRISPR) revealing the presence of four to seven arrays with multiple spacers: seven arrays, containing a total of 24 spacers, were identified in *Limnothrix* sp. CACIAM 69d; five arrays and 57 total spacers are present in *Limnothrix* sp. PR1529; four arrays were identified in *Limnothrix* sp. P13C2 (66 total spacers) and *Limnothrix rosea* IAM M-220 (316 spacers). Alignments of the spacers and their potential targets can be found in Supplementary Figures 1–4.

**FIGURE 4:**
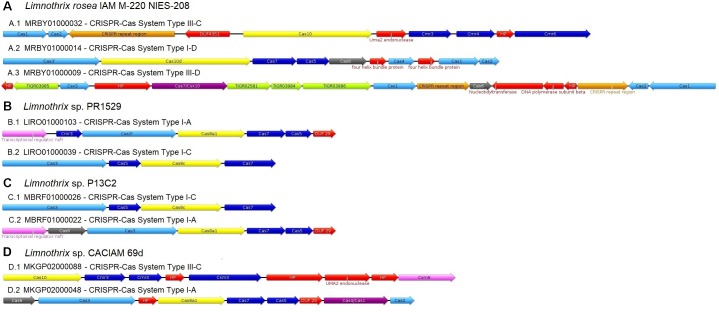
Classification of CRISPR-Cas systems found in all four analyzed *Limnothrix* genome **(A–D)**. The colors indicate the following categories: repeat-associated mysterious protein (RAMP) family RNases involved in crRNA process (gray), CRISPR-Cas protein with no major category (light blue), large CRISPR-associated complex for antiviral defense (Cascade) subunits (yellow), RAMP Cascade subunits (dark blue), fused Cas protein (purple), transcriptional regulator (light pink), protein containing a CRISPR-associated domain (green), CRISPR repeat region (orange) and domain of unknown function (DUF) or hypothetical protein (HP) or other (red).

The CRISPR-Cas systems identified in *Limnothrix* genomes were classified according to the classification criteria most often adopted (Makarova et al., 2015) and are shown in Figure 4. With exception of *Limnothrix rosea* IAM M-220, only two CRISPR-Cas systems were identified in all *Limnothrix*, one of them classified as type I-A. Besides that, both *Limnothrix* sp. P13C2 and *Limnothrix* sp. PR1529 showed identical results in CRISPR-Cas system classification and gene order.

### General Metabolism

**FIGURE 5:**
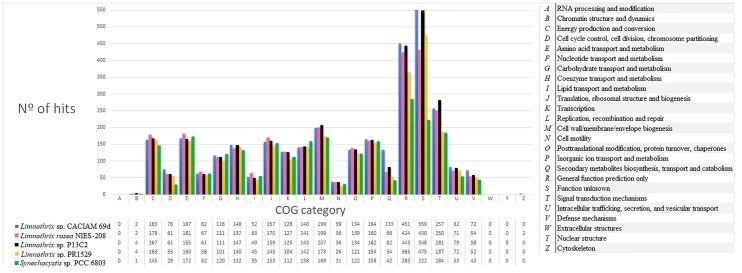
Number of hits per COG category for all four *Limnothrix* genomes and *Synechocystis* sp. PCC 6803.

Clusters of Orthologous Group (COG) analyses have been performed on cyanobacterial genomes to obtain their functional profiling (Prabha et al., 2016), study cyanobacteria metabolism via comparative genomics (Leao et al., 2017) and to obtain the composition features of their core genomes (Simm et al., 2015). In this way, to gain further understanding of the general *Limnothrix* metabolism, a COG comparison was performed between all four *Limnothrix* genomes and *Synechocystis* sp. PCC 6803, which is a cyanobacterial model for several studies (Figure 5). Relevant differences were observed; strains CACIAM 69d, P13C2 and *Limnothrix rosea* IAM M-220 presented more genes related to cell wall/membrane/envelope biogenesis (M) than PCC 6803 and PR1529. Moreover, the number of hits in categories R (general function prediction only) and S (function unknown) are higher in *Limnothrix* in comparison to *Synechocystis* sp. PCC 6803. Remarkably, the strains CACIAM 69d and P13C2 had a number of hits greater than 500 for category S.

**FIGURE 6:**
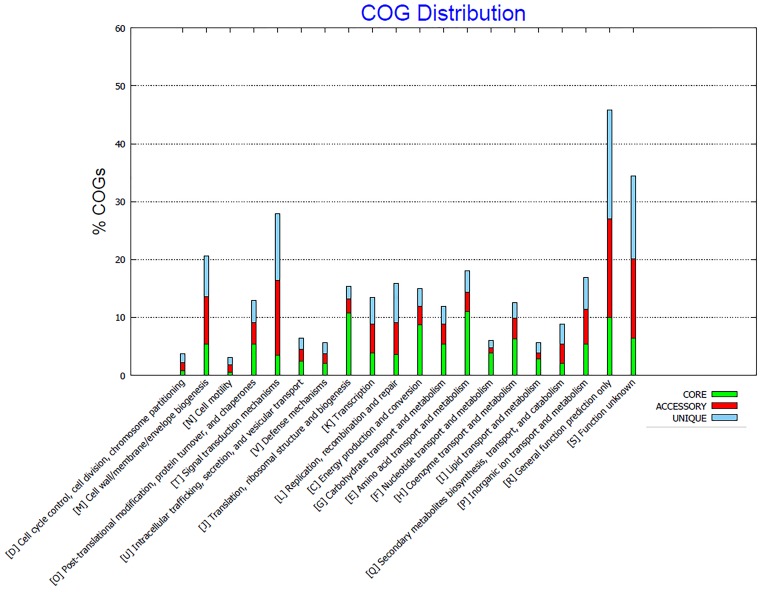
COG distribution of core, accessory and unique genes present in all four analyzed *Limnothrix* genomes generated by Bacterial Pan Genome Analysis Tool (BPGA).

In a pan-genome analysis, the core genome is the set of genes shared by all strains, being responsible for major phenotypic traits and basic metabolism of the analyzed group (Chaudhari et al., 2016). In this way, the four analyzed *Limnothrix* strains share approximately 30% of the total coding CDS content (Table 2), most of them related to energy metabolism (Figure 6). There are as well many accessory genes, i.e., dispensable genes that occur in two or more strains (Chaudhari et al., 2016), present in CACIAM 69d, P13C2 and PR1529. Due to the close phylogenetic relation of these strains (Figures 2, 3), we hypothesized that many of these accessory genes are shared among them, since *Limnothrix rosea* IAM M-220 presented a high number (64.8%) of unique genes (Table 2). A new pan-genome analysis excluding *Limnothrix rosea* IAM M-220 revealed a different core genome size, with approximately 74% of total CDS content (Table 3). Thus, many accessory genes present in strains CACIAM 69d, P13C2 and PR1529 showed in Table 2 (the first analysis) were grouped in the core genome in the second analysis (Table 3), since the number of unique genes did not vary much in both investigations. Additional pan-genome analysis of *Limnothrix rosea* IAM M-220 (Supplementary Tables 3–6) revealed that it shares 77% of its genes with *Leptolyngbya* sp. PCC 7376; the latter shares 64.5% with this *Limnothrix*. The differences between *Limnothrix rosea* IAM M-220 and the three other *Limnothrix* strains became clearer when some individual aspects of the genomes were analyzed, as further discussed below.

**Table 2:** Number of protein sequences for representatives of core, accessory and unique orthologous clusters present in all four *Limnothrix* genomes.

Organism	No. of core genes	No. of accessory genes	No. of unique genes	No. of coding CDS
*Limnothrix rosea* IAM M-220	1,088	81	2,296	3,540
*Limnothrix* sp. CACIAM 69d	1,088	2,008	451	3,718
*Limnothrix* sp. P13C2	1,088	2,417	59	3,672
*Limnothrix* sp. PR1529	1,088	2,155	68	3,723


**Table 3:** Number of protein sequences for representatives of core, accessory and unique orthologous clusters present in *Limnothrix* genomes, excluding *Limnothrix rosea* IAM M-220.

Organism	No. of core genes	No. of accessory genes	No. of unique genes	No. of coding CDS
*Limnothrix* sp. CACIAM 69d	2,741	303	496	3,718
*Limnothrix* sp. P13C2	2,741	759	59	3,672
*Limnothrix* sp. PR1529	2,741	498	69	3,723


Comparing the COG distribution of the genetic content present in *Limnothrix* genomes, it was possible to identify conserved categories associated with the core genome, such as energy production and conversion (C), carbohydrate (G), amino acid (E), nucleotide (F), coenzyme (H), and lipid (I) transport and metabolisms (Figure 6). On the other hand, some categories, such as M, S and R have a high percentage of accessory and unique genes. The exclusion of *Limnothrix rosea* IAM M-220 from this analysis revealed a decrease of unique and accessory COGs in category C. Furthermore, no unique COGs were identified in category I (Supplementary Figure 5).

**FIGURE 7:**
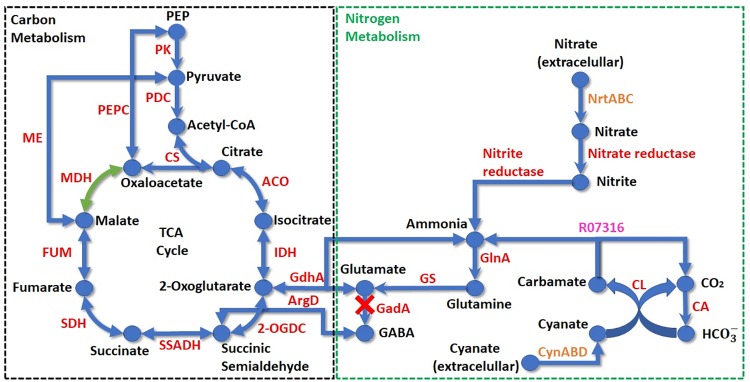
Schematic showing TCA cycle and nitrogen acquisition pathways in *Limnothrix*. The dotted boxes delimit carbon (black) and nitrogen (green) metabolisms. Compounds are represented by circles and their respective names indicated in black. Arrows indicate reactions; enzyme reactions are shown in red, transport reactions in orange and a spontaneous KEGG reaction is shown in purple. The green reaction occurs exclusively in *Limnothrix rosea* IAM M-220 NIES-208. ACO, aconitase; ArgD, γ-aminobutyric acid aminotransferase; CA, carbonic anhydrase; CL, cyanate lyase; CS, citrate synthase; FUM, fumarase; GadA, glutamate decarboxylase; GdhA, glutamate dehydrogenase; GlnA, glutamine synthase; GS, glutamine synthase; IDH, isocitrate dehydrogenase; MDH, malate dehydrogenase; ME, malic enzyme; 2-OGDC, 2-oxoglutarate decarboxylase; PDC, pyruvate dehydrogenase complex; PEP, phosphoenolpyruvate; PEPC, phosphoenolpyruvate carboxylase; PK, pyruvate kinase; SDH, succinate dehydrogenase; SSADH, succinic semialdehyde dehydrogenase.

Despite the central carbon metabolism, the complete pathways of glycolysis, Calvin-Benson cycle and pentose phosphate were identified and all core enzymes of the cyanobacterial pyruvate metabolism (Beck et al., 2012) were present in *Limnothrix* genomes analyzed here. The tricarboxylic acid (TCA) cycle is completed through conversion of 2-oxoglutarate to succinic semialdehyde, by 2-oxoglutarate decarboxylase, and then to succinate by a succinic semialdehyde dehydrogenase (Figure 7). The cyanobacterial glyoxylate shunt is not present in *Limnothrix* and the γ-aminobutyrate (GABA) shunt is incomplete due to the absence of glutamate decarboxylase (GadA). *Limnothrix rosea* IAM M-220 is the only genome that has malate dehydrogenase (EC 1.1.1.37). The malic enzyme (EC 1.1.1.38) compensates for the lack for malate dehydrogenase in strains CACIAM 69d, PR1529 and P13C2, converting malate to pyruvate; it is also present in *Limnothrix rosea* IAM M-220, acting as an alternative pathway for obtaining pyruvate.

The MEP (methylerythritol-phosphate) pathway, which uses glyceraldehyde 3-phosphate and pyruvate to synthesize terpenoids (Pattanaik and Lindberg, 2015), is present in all four *Limnothrix*. Additionally, the CACIAM 69d, P13C2 and PR1529 strains are capable of producing hydrocarbons from fatty acids using the AAR/ADO (acyl-ACP reductase/aldehyde-deformylating oxygenase) pathway, while *Limnothrix rosea* IAM M-220 appears to achieve the same using the OLS (α-olefin synthase) pathway (Zhu et al., 2018), which involves the participation of PKS modules (Coates et al., 2014).

In the carbon fixation pathway of photosynthetic organisms, a difference in erythrose-4P synthesis was observed between all *Limnothrix* and *Synechocystis* sp. PCC 6803: the former has glycoaldehydetransferase (EC 2.2.1.1), which uses D-fructose 6P and glyceraldehyde-3P, producing xylulose-5P, while the latter has fructose-phosphate phosphoketolase (EC 4.1.2.22), which acts on D-fructose 6P and phosphate. Regarding the storage metabolism of *Limnothrix*, genes involved in synthesis and mobilization of glycogen and cyanophycin were found in the four genomes.

Analysis of the nitrogen metabolism revealed that *Limnothrix* can use nitrate and cyanate as nitrogen sources (Figure 7), and all genomes lack the genes for nitrogenases. Nitrogen and carbon metabolisms are connected through ammonia by glutamate dehydrogenase, which produces 2-oxoglutarate for the TCA cycle.

Further analysis revealed that the strains CACIAM 69d, PR1529 and P13C2 can use organosulfonated compounds to acquire sulfur under limiting conditions due to presence of the *ssu* operon in their genomes (Eichhorn et al., 2000; Ellis, 2011). Besides that, those strains can also incorporate sulfur in their metabolism by using thiosulfate as substrate for thiosulfate sulfurtransferase (EC 2.8.1.1).

### Genome Mining

Cyanobacteria harbors genes for three main pathways related to assembly and export of extracellular polymeric substances: Wzy-, ABC transporter- and Synthase dependent (Pereira et al., 2015). A search for protein domains present in those mechanisms revealed similar results for all *Limnothrix* (Supplementary Table 7). In this way, there is a functional Wzy pathway, along with some identified components of ABC transporter (KpsD, KpsE, KpsM, KpsT, and KpsU) and Synthase dependent (Alg8/BcsA, AlgG, AlgI and ExoD) pathways, with multiple results for Alg8/BcsA.

**FIGURE 8:**
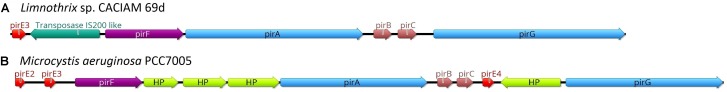
Piricyclamide gene cluster schematics. **(A)** Cluster found in *Limnothrix* sp. CACIAM 69d contig MKGP02000097 and **(B)** cluster previously described in *Microcystis aeruginosa* PCC7005. The precursor genes are shown in red, proteases are blue, prenyltransferase is purple, genes with no prediction function are brown, hypothetical proteins (HP) are light green, a transposase is dark green, and R is for repeat region.

The antiSMASH tool (Blin et al., 2017) detected a piricyclamide gene cluster in *Limnothrix* sp. CACIAM 69d genome, which was previously described only in the genus *Microcystis* (Leikoski et al., 2012). Manual curation pointed to the location of one precursor gene (pirE3) in contig MKGP02000097 (Figure 8A). Additional searches performed by HMMER 3 (Eddy, 2011) using TIGR04446 (prenylated cyclic peptide, anacyclamide/piricyclamide family) against all CACIAM 69d CDS returned no results other than the previously identified pirE3. In comparison to *Microcystis aeruginosa* PCC7005 (Figure 8B), *Limnothrix* sp. CACIAM 69d presents: (i) no hypothetical proteins; (ii) a transposase IS200 like (PF01797) inserted between pirF and a precursor gene pirE3; and (iii) only one precursor gene pirE.

**FIGURE 9:**
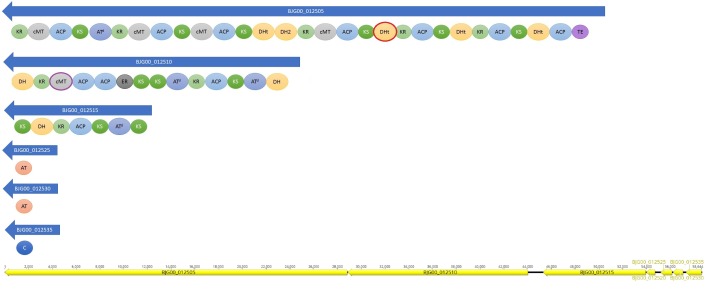
Domains present in *trans*-AT PKS cluster found in *Limnothrix* sp. CACIAM 69d and *Limnothrix* sp. P13C2. The results are presented according to antiSMASH 4.0 output. The red circle indicates a domain present only in P13C2 strain and the purple circle a domain found only in CACIAM 69d strain. The order of the CDS for CACIAM 69d and P13C2 is shown at the bottom. ACP, acyl carrier protein; AT, acyltransferase domain; C, condensation domain; DH, dehydratase domain; ER, enoyl reductase domain; KR, keto reductase domain; KS, keto synthase domain; MT, methyl transferase domain; TE, thioesterase domain.

**FIGURE 10:**
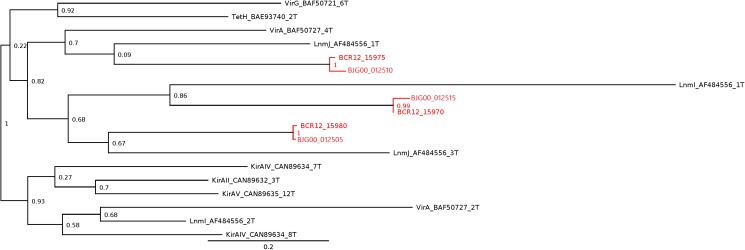
Subtree generated by NaPDoS for *Limnothrix* sp. CACIAM 69d and *Limnothrix* sp. P13C2 *trans*-AT PKS cluster analysis. *Limnothrix* domain sequences are in red and named based on Table 4; the initials BCR12 and BJG00 are, respectively, from *Limnothrix* sp. P13C2 and *Limnothrix* sp. CACIAM 69d. Other domain sequences are named according to the NaPDoS database: Vir, virginiamycin; Tet, tetronomycin; Lnm, leinamycin; Kir, kirromycin. The numbers presented are confidence values generated in the NaPDoS tree reconstruction process, which uses the FastTree (Guindon and Gascuel, 2003) to estimate maximum likelihood. The complete tree is available in Supplementary File 3.

A *trans*-AT PKS cluster was detected in contig MKGP02000067 of *Limnothrix* sp. CACIAM 69d and contig MBRF01000028 of *Limnothrix* sp. P13C2, both showing similar module compositions (Figure 9). A NaPDoS (Ziemert et al., 2012) analysis for its KS domain containing proteins returned hits for domains present in the genes responsible for the biosynthesis of virginiamycin, leinamycin and kirromycin (Table 4). Despite showing higher identity for virginiamycin and kirromycin, the proteins clustered together with genes related to biosynthesis of leinamycin, LnmI and LnmJ (Figure 10). However, they displayed a long phylogenetic distance from the related genes.

**Table 4:** NaPDoS results for KS domain containing proteins present in *trans*-AT PKS clusters found in *Limnothrix* sp. CACIAM 69d and *Limnothrix* sp. P13C2.

Query ID (locus tag)	NaPDoS database match ID	Percent identity	Align length	E-value	Pathway product	Organism
BJG00_012525	KirAIV_CAN89634_7T	55	417	7 E-117	Kirromycin	*Limnothrix* sp. CACIAM 69d
BJG00_012510	VirA_BAF50727_4T	59	438	2 E-138	Virginiamycin	
BJG00_012505	VirA_BAF50727_4T	57	431	5 E-133	Virginiamycin	
BCR12_15970	KirAIV_CAN89634_7T	55	426	5 E-120	Kirromycin	*Limnothrix* sp. P13C2
BCR12_15975	LnmJ_AF484556_1T	59	432	6 E-137	Leinamycin	
BCR12_15980	VirA_BAF50727_4T	57	431	1 E-133	Virginiamycin	


Additional searches with BLAST in *Limnothrix* genomes returned no results for anatoxin, cylindrospermopsin, lyngbyatoxin, microcystin, nodularin and saxitoxin gene clusters.

## Discussion

Genome assembly using different algorithms followed by a comparison binning process was capable of reconstructing a nearly complete and representative uncontaminated genome of *Limnothrix* sp. CACIAM 69d, which is phylogenetically close to *Limnothrix* sp. CENA545 from a Brazilian Pantanal saline-alkaline lake (Andreote et al., 2014) (Figure 2). Thus, it was possible to analyze the actual gene content in order to reveal important aspects of this cyanobacterial metabolism, both central and secondary. Several cyanobacterial genomes display a high number of repeats due to insertion sequences (Vigil-Stenman et al., 2015), which can be a problem because assembly algorithms cannot characterize repeat elements that span longer than read length (Alkan et al., 2010). Nevertheless, the assembly of some regions could have been affected by repetition sequences, which may have prevented the formation of longer contigs. A potential solution for this problem is to increase read length using new technologies such as PacBio or Oxford Nanopore MinION (Koren and Phillippy, 2015).

Regarding *Limnothrix rosea* IAM M-220, its position within both phylogenetic trees (Figures 2, 3) raised the suspicions as to whether this strain really belongs to the *Limnothrix* genus. This strain was originally named *Oscillatoria rosea* NIES-208 when the genome was published by Zhu et al. (2017). However, the GenBank record (MRBY00000000) provided by that work points to *Limnothrix rosea* IAM M-220. Furthermore, the NCBI Taxonomy Browser lists *Oscillatoria rosea* NIES-208 as a synonym for *Limnothrix rosea* IAM M-220 (Taxonomy ID: 454133). A comparison of *Limnothrix rosea* IAM M-220 and three other available *Oscillatoria* genomes (Supplementary Table 5) revealed that they share only 961 genes, which is only 19.5% of the former total coding CDS. An additional comparison of *Limnothrix rosea* IAM M-220 and other two *Spirulina* genomes also resulted in a low number of shared genes: 1,313, representing 37% of the former total coding CDS (Supplementary Table 6). This last value, however, is higher than the one presented in Table 2, which considers the three other *Limnothrix* genomes. Based on the Species Tree generated by KBase (Figure 3), we have assumed that perhaps *Limnothrix rosea* IAM M-220 was close to the genus *Leptolyngbya*, which is also from the Cyanobacteria Subsection III (Castenholz et al., 2015a), since it shares 77% of its genes with *Leptolyngbya* sp. PCC 7376 (Supplementary Table 3). Nevertheless, this hypothesis was discarded when another pan-genome analysis was performed adding other genomes of *Leptolyngbya* returned none core genes (data not shown). *Limnothrix rosea* IAM M-220 has 1,002 genes shared with some strains of *Leptolyngbya* (PCC 7376, PCC 6406 and O 77) in a pan-genome analysis (Supplementary Table 4). Given these data, we were not able to determinate if *Limnothrix rosea* IAM M-220 belongs to the genus *Leptolyngbya* and concluded that this strain is not a *Limnothrix*. We even raise suspicion regarding the *Leptolyngbya* sp. PCC 7376 taxonomic classification. In this sense, only a polyphasic approach could be able to correctly classify these cyanobacteria taxonomically (Komárek et al., 2014).

Many bacteria and most archaea have an adaptive immune system known as CRISPR-Cas. The CRISPR locus contain a sequence array, comprising short direct repeats separated by short variable DNA sequences, which are called spacers. Aided by Cas protein, the spacers specifically target invading virus or plasmid sequences (i.e., the protospacers) and cleave their nucleic acids. Thus, they are responsible for the sequence memory of this immune system (Makarova et al., 2015). Regarding the CRISPR search in *Limnothrix* genomes, many spacer sequences were found, but only a few of them had potential matches (protospacers) when compared to GenBank-phage and plasmid RefSeq databases (Supplementary Figures 1–4). For strains CACIAM 69d and PR1529, about 12.5% and 9% of spacers, respectively, had potential targets; about 11% and 4% spacers from, respectively, strain P13C2 and *Limnothrix rosea* IAM M-220. Most of the protospacers sequences found belong to plasmids and are probably unrelated to cyanobacteria. Cyanophage protospacers were only found in *Limnothrix rosea* IAM M-220, and yet they are related to *Synechococcus* phages (Supplementary Figure 4). Cyanophages are important agents in cyanobacterial population control and evolution, since they can transfer genes among cyanobacteria and also act as an environmental gene storage (Shestakova and Karbysheva, 2015). The fact that most of the spacers present in *Limnothrix* genomes have no known targets indicates the lack of representation of these cyanophages in public databases. This could be due to scarce genomic data available from these *Limnothrix* environments.

Cai et al. (2013) addressed the distribution of CRISPR-Cas systems in cyanobacteria and found that, in general, subsection III has a median of about 100 spacer sequences and tends to have a large number of them, with a maximum around 600 sequences. Considering the results found in *Limnothrix* (subsection III), only *Limnothrix rosea* IAM M-220 presented a high number of spacer sequences, which could be related to the presence of Cas1 and Cas2 in its CRISPR-Cas systems (Figure 4A). These two proteins are responsible for forming an adaptation module complex required for the insertion of spacers into CRISPR arrays (Makarova et al., 2015). Thus, when comparing the number of spacers in *Limnothrix rosea* IAM M-220 genome with the other three *Limnothrix* genomes, which CRISPR-Cas systems lack Cas1 and Cas2 (Figures 4B–D), it is expected that the former will have more spacers due to Cas1 and Cas2 function. However, it is possible that these three *Limnothrix* genomes have another way to acquire new protospacers via a mechanism yet to be discovered. Since the incorporation of new spacers and the loss of existing spacer sequences is related to a highly dynamic response to the environment (Kuno et al., 2012), another explanation is that there is not enough pressure in their environment to acquire new sequences; or there is pressure for them to lose their previously acquired spacers.

Regarding the central carbon metabolism, all *Limnothrix* genomes and *Synechocystis* sp. PCC 6803 investigated have the key enzymes, highlighting that the former (except for *Limnothrix rosea* IAM M-220) are better equipped for environmental sulfur acquisition in limiting conditions. Inorganic sulfate is the second most abundant ion present in aquatic environments, acting as a main source of sulfur for microorganisms (Loka Bharathi, 2008). However, under sulfur-limiting conditions, bacteria are capable of using organosulfonates as sulfur sources (Ellis, 2011). Due to the *ssu* operon, *Limnothrix* sp. CACIAM 69d is able to acquire sulfur from organic substrates, such as C-2 to C-10 alkanesulfonates (Eichhorn et al., 1999), anaerobically released from the bottom of the lake (Pereira et al., 1994; Fonseca et al., 2012), thus adapting to the seasonal changes affecting sulfate levels on Tucuruí Hydroelectric Reservoir. *Limnothrix* sp. P13C2 may face similar sulfate limitations in Pandan Reservoir (1°18′51.8″ N, 103°44′29.2″ E), Singapore, as a spatiotemporal analysis of waterways flowing from this reservoir showed different levels of sulfate along the water course (Saxena et al., 2015).

*Limnothrix* genomes harbor transporters for nitrate, ammonium and cyanate, indicating that they can use these compounds as nitrogen sources. Due to rainfall changes, the concentration of nitrate is affected in Tucuruí Hydroelectric Reservoir (Brandão et al., 2017), reaching low levels at the water surface in dry periods (Baraúna et al., 2013). This suggests that ammonium may be used as the primary nitrogen source during low rain season. Nonetheless, the presence of cyanate transporter, allied with a higher density of rotifers (zooplankton) in the dry period (Francineli et al., 2015), could indicate that cyanate has an important role in *Limnothrix* nitrogen metabolism. It is known that zooplankton can excrete nutrients to support primary production in lakes (Vanni, 2002). In this way, the excreted urea can undergo spontaneous dissociation to produce cyanate (Kamennaya et al., 2008), which can be acquired by *Limnothrix* and then converted to ammonium (Figure 7). Additionally, urea can also be supplied by the decomposing organic matter at the bottom of the lake. Even if there is not enough nitrogen available, *Limnothrix* is able to overcome this using the stored cyanophycin, which is a polymer composed of aspartate and arginine, as nitrogen and carbon source (Beck et al., 2012).

Cyanobacteria have an universal ability to produce hydrocarbons (Han et al., 1968, 1969; Winters et al., 1969), which can be achieved via AAR/ADO and OLS pathways. Naturally, these pathways do not occur in the same organism, with the former presenting the largest taxonomical distribution (Coates et al., 2014). Since the AAR/ADO pathway was found only in strains CACIAM 69d, P13C2 and PR1529, we suppose that *Limnothrix rosea* IAM M-220 possesses the OLS pathway. A closer look at contig MRBY01000033 from the latter revealed a beta-ketoacyl synthase (NIES208_12590) that has the same domains when compared to OLS PKS modules that were presented by Coates et al. (2014). A recent study also reported the presence of an OLS pathway in *Limnothrix rosea* IAM M-220 using bioinformatic analyses (Zhu et al., 2018).

The production of many EPS by *Limnothrix redekei* PUPCCC 16 has been reported, and most of them are released into the cultivation medium, making the isolation process easy (Khattar et al., 2010). Given the high number of genes present in the COG category cell wall/membrane/envelope biogenesis (Figure 5) and multiple hits for Alg8/BcsA (Supplementary Table 7), we supposed that *Limnothrix* can produce a broad variety of extracellular polymeric substances that could be EPS. In a previous study (Pereira et al., 2015), AlgG was detected in a very restricted number of cyanobacterial strains and the absence of AlgI was observed in *Synechocystis* sp. PCC 6803. These enzymes are involved in the synthesis of alginate (Franklin et al., 2011), an EPS that confers a mucoid phenotype (Evans and Linker, 1973). However, the other enzymes required for alginate biosynthesis were not found in *Limnothrix* genomes (Supplementary Table 7). This investigation of these differently sequenced and assembled genomes suggests that the full mechanism of alginate biosynthesis may be not truly present in *Limnothrix*. Furthermore, it is speculated that EPS production in cyanobacteria may not follow existing patterns (Pereira et al., 2015). Therefore, more *Limnothrix* genomes are needed to solve this question.

Only recently, have some studies begun investigating the toxicity of *Limnothrix* (Bernard et al., 2011; Humpage et al., 2012; Daniels et al., 2014). These works have not detected the presence of previously described cyanobacterial toxins (microcystins, saxitoxins, and cylindrospermopsins). BLAST searches in *Limnothrix* genomes returned no results for the aforementioned toxins and were also negative for anatoxin and lyngbyatoxin. A novel toxin called Limnothrixin, which has been associated to damage in liver, lungs and gastrointestinal tract of mice (Humpage et al., 2012) as well as toxic activity against *Bufo marinus* larvae (Daniels et al., 2014), has been proposed. However, since there is no resolved structure for this toxin, the identification of genes responsible for its biosynthesis is difficult.

Cyanobacterial toxins have different chemical structures (Bláha et al., 2009) (peptides, alkaloids, lipidic compounds and lipopolysaccharides), which can be produced by a variety of mechanisms and pathways (Dittmann et al., 2013). The *trans*-AT PKS gene cluster detected in both *Limnothrix* sp. CACIAM 69d and *Limnothrix* sp. P13C2 may be a potential candidate for Limnothrixin biosynthesis, since it appears to be conserved among the genomes (Figure 9), presenting a long phylogenetic distance (Figure 10) and less than 80% identity with other known compounds present in the NaPDoS database; moreover, the *trans*-AT PKS systems are capable of producing many structurally complex, bioactive natural compounds (Helfrich and Piel, 2016).

Cyanobactins are small cyclic peptides ribosomally produced by many cyanobacteria using solely proteinogenic amino acids (Sivonen et al., 2010). Piricyclamide is a cyanobactin previously identified in strains of *Microcystis* (Leikoski et al., 2012), but no toxicity assays have been performed on it so far. The cluster identified in *Limnothrix* sp. CACIAM 69d (Figure 8A) has one precursor gene, and although it was not possible to find other precursor genes, they could be located in an unsequenced and/or unassembled region of the genome. Since cyanobactins have a broad toxic activity (Sivonen et al., 2010), it is possible that they may contribute, along with lipopolysaccharides, to *Limnothrix* toxicity.

Although only four genomes are currently available for the genus *Limnothrix*, it was possible to analyze their metabolic diversity, which is a key feature present in Cyanobacteria phylum. The draft genome of *Limnothrix* sp. CACIAM 69d adds a new member to the small *Limnothrix* genome library and contributes to a growing representativeness of cyanobacterial genomes from Amazon region. The comparative genomic analysis of *Limnothrix* made it possible to highlight unique genes for each strain and to understand the overall features of their metabolism. Furthermore, it was important to show inconsistencies in cyanobacteria taxonomical classification of *Limnothrix*.

## Author Contributions

AL, AS, JA, DA, PM, LD, and EG designed the project and the analysis procedures. AS collected, cultivated, and extracted the DNA from the sample. CdL performed the sequencing protocol. AL and JP executed the bioinformatics tools and pipelines. AL, AS, JV, PM, LX, LD, and EG analyzed the final data and wrote the manuscript. DA, JV-J, MN, and EG contributed with materials, reagents, and equipment. All the authors read and approved the final version of the manuscript.

## Conflict of Interest Statement

The authors declare that the research was conducted in the absence of any commercial or financial relationships that could be construed as a potential conflict of interest.The reviewer AL and handling Editor declared their shared affiliation.
