# Supplementary material for: Insights Into Limnothrix sp. Metabolism Based on Comparative Genomics
Source: Front Microbiol. 2018 Nov 20;9:2811. doi: 10.3389/fmicb.2018.02811 (PMC6256058; doi:10.3389/fmicb.2018.02811)
Supplement: Supplementary file 1 [file Data_Sheet_1.ZIP › Newbler_comparisons/quast_results/results_2018_08_03_15_28_44/report.pdf]

Report

	bin.001_Cyano	bin.5_Cyano	Cluster.8_Cyano
# contigs (>= 0 bp)	104	90	122
# contigs (>= 1000 bp)	99	90	114
# contigs (>= 5000 bp)	90	87	90
# contigs (>= 10000 bp)	76	75	76
# contigs (>= 25000 bp)	55	55	55
# contigs (>= 50000 bp)	33	33	33
Total length (>= 0 bp)	4570527	4528652	4597952
Total length (>= 1000 bp)	4566969	4528652	4592401
Total length (>= 5000 bp)	4545757	4518218	4545711
Total length (>= 10000 bp)	4446663	4429981	4446663
Total length (>= 25000 bp)	4108984	4108984	4108984
Total length (>= 50000 bp)	3303677	3303677	3303677
# contigs	104	90	122
Largest contig	241377	241377	241377
Total length	4570527	4528652	4597952
GC (%)	55.24	55.27	55.21
N50	86879	90235	86879
N75	46876	47462	45306
L50	18	17	18
L75	36	35	37
# N's per 100 kbp	0.02	0.00	0.13

All statistics are based on contigs of size >= 500 bp, unless otherwise noted (e.g., "# contigs (>= 0 bp)" and "Total length (>= 0 bp)" include all contigs).

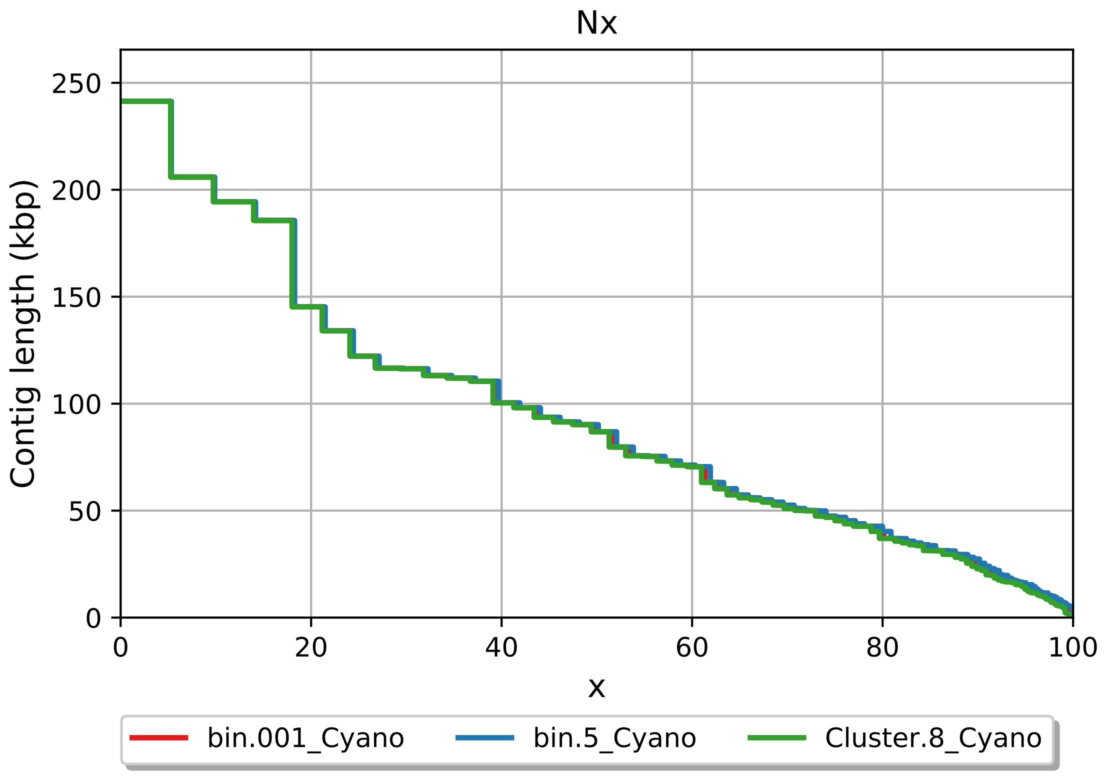

Nx
Contig length (kbp)
250
200
150
100
50
0
0
20
40
60
80
100
x
bin.001_Cyano
bin.5_Cyano
Cluster.8_Cyano

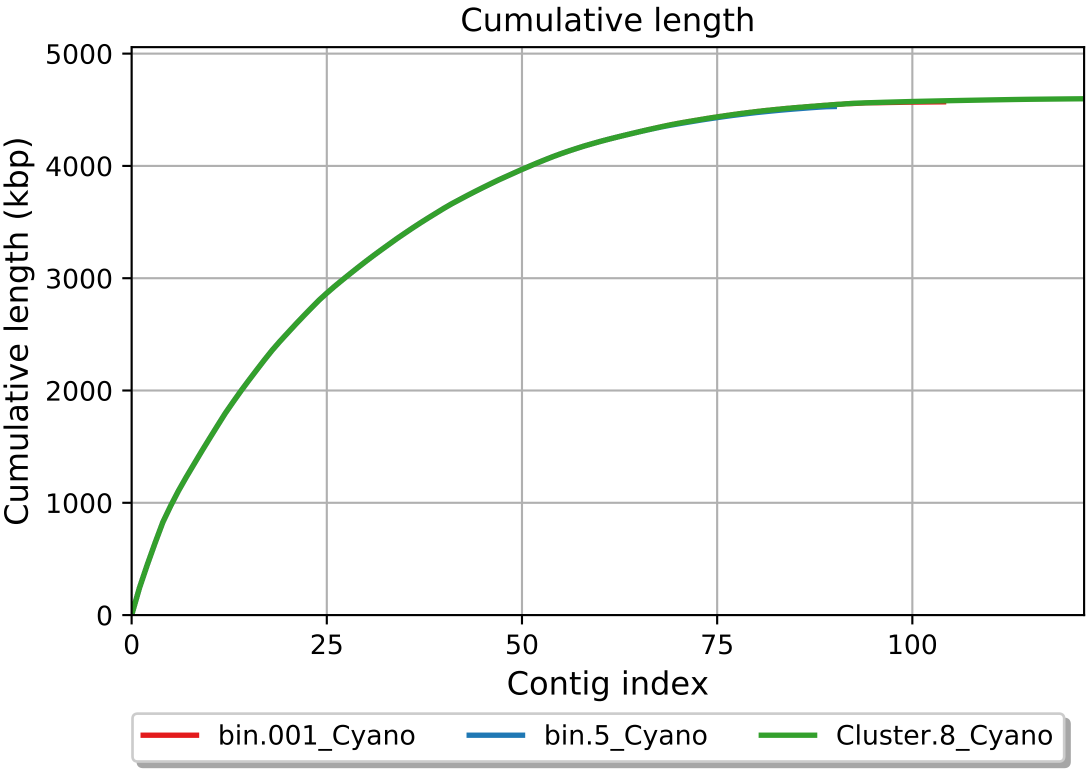

Cumulative length
Cumulative length (kbp)
0
1000
2000
3000
4000
5000
0
25
50
75
100
Contig index
bin.001_Cyano
bin.5_Cyano
Cluster.8_Cyano

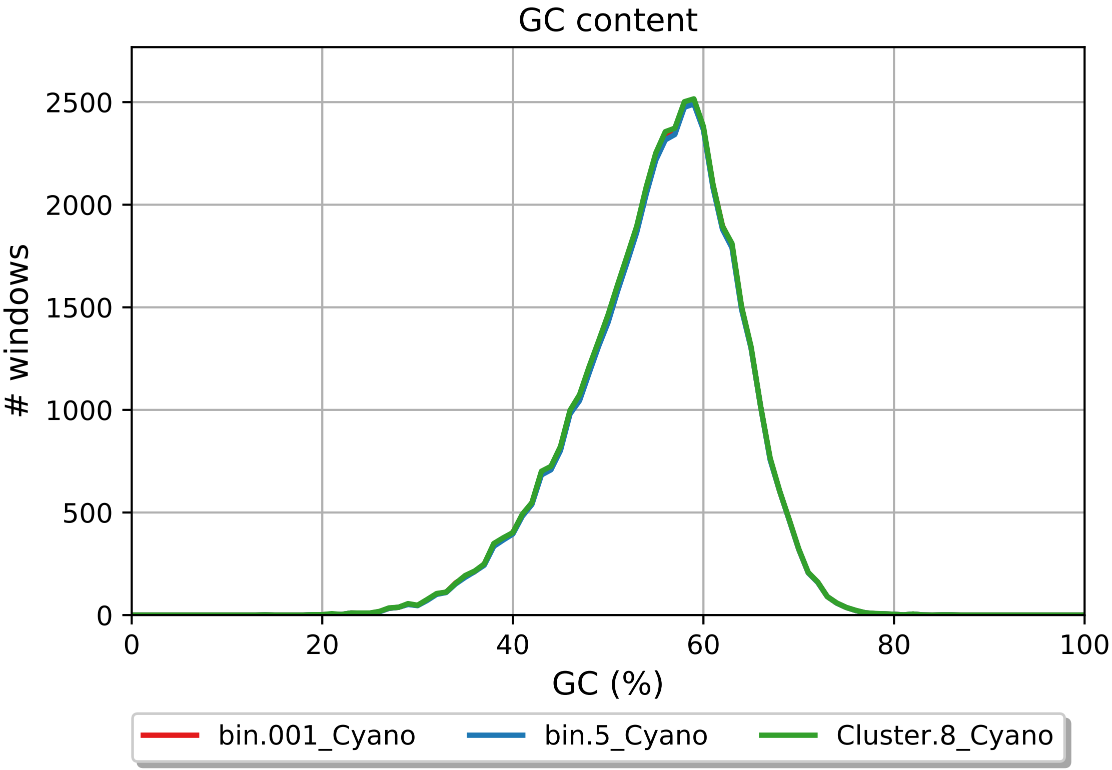
GC content
windows
0
500
1000
1500
2000
2500
0
20
40
60
80
100
GC (%)
bin.001_Cyano
bin.5_Cyano
Cluster.8_Cyano

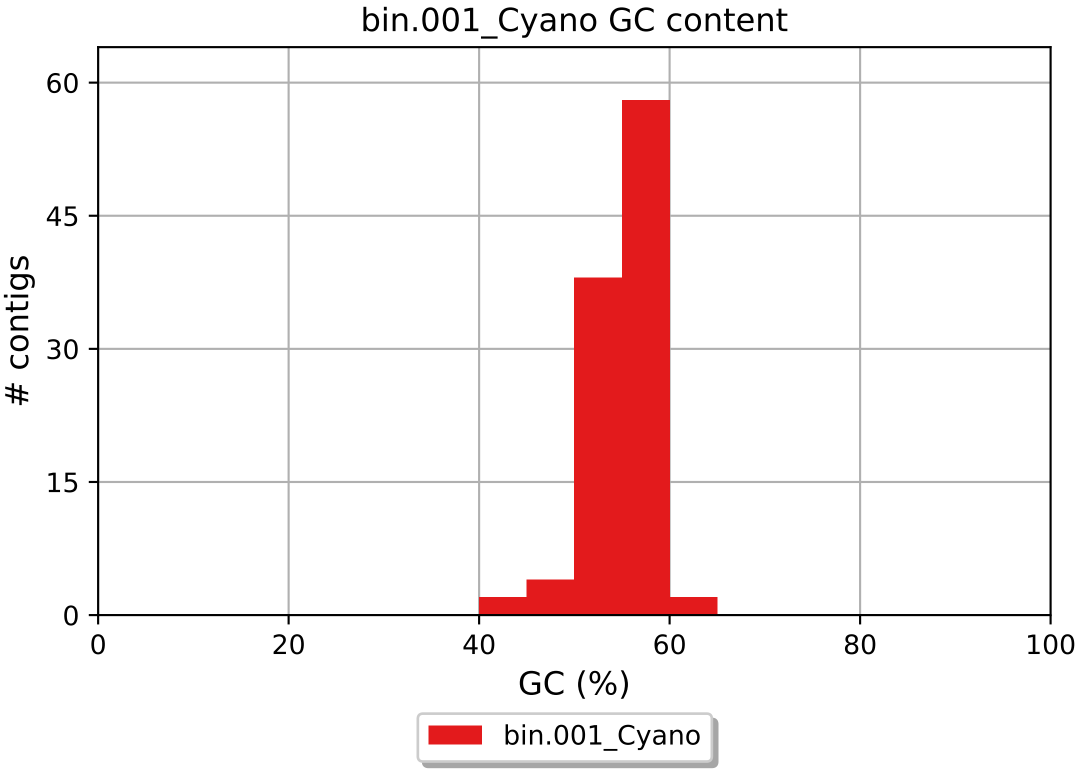
bin.001_Cyano GC content
60
45
30
15
0
contigs
0
20
40
60
80
100
GC (%)
bin.001_Cyano

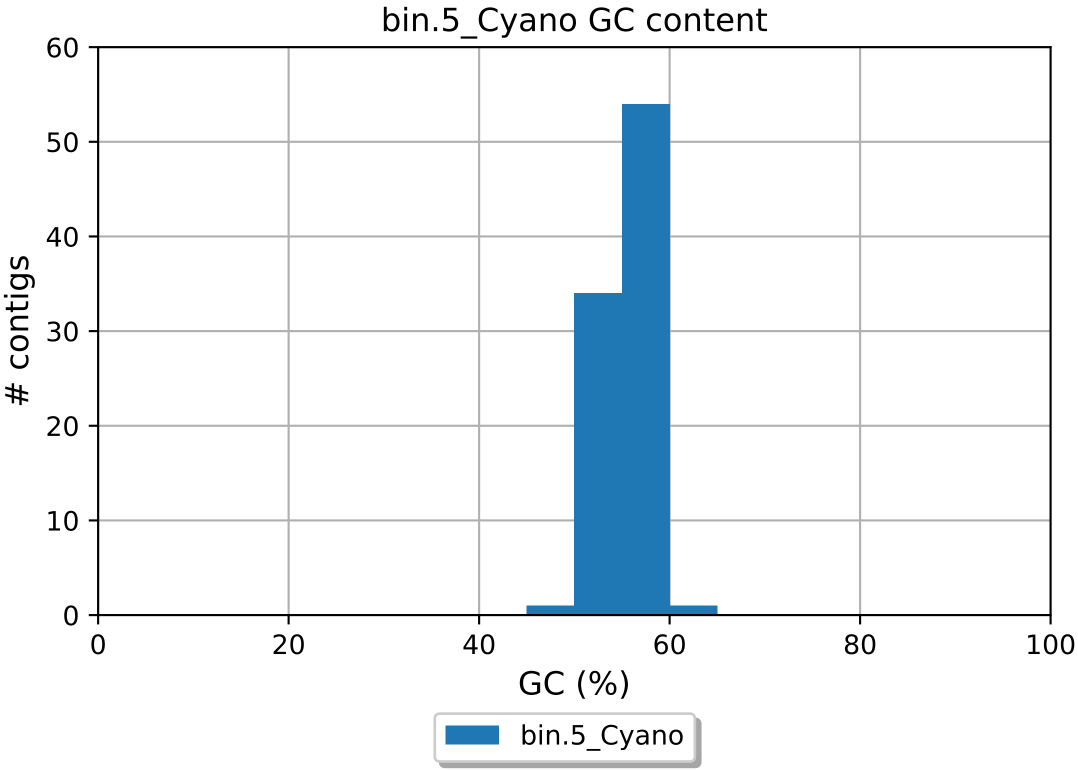
bin.5_Cyano GC content
60
50
40
30
20
10
0
contigs
0
20
40
60
80
100
GC (%)
bin.5_Cyano

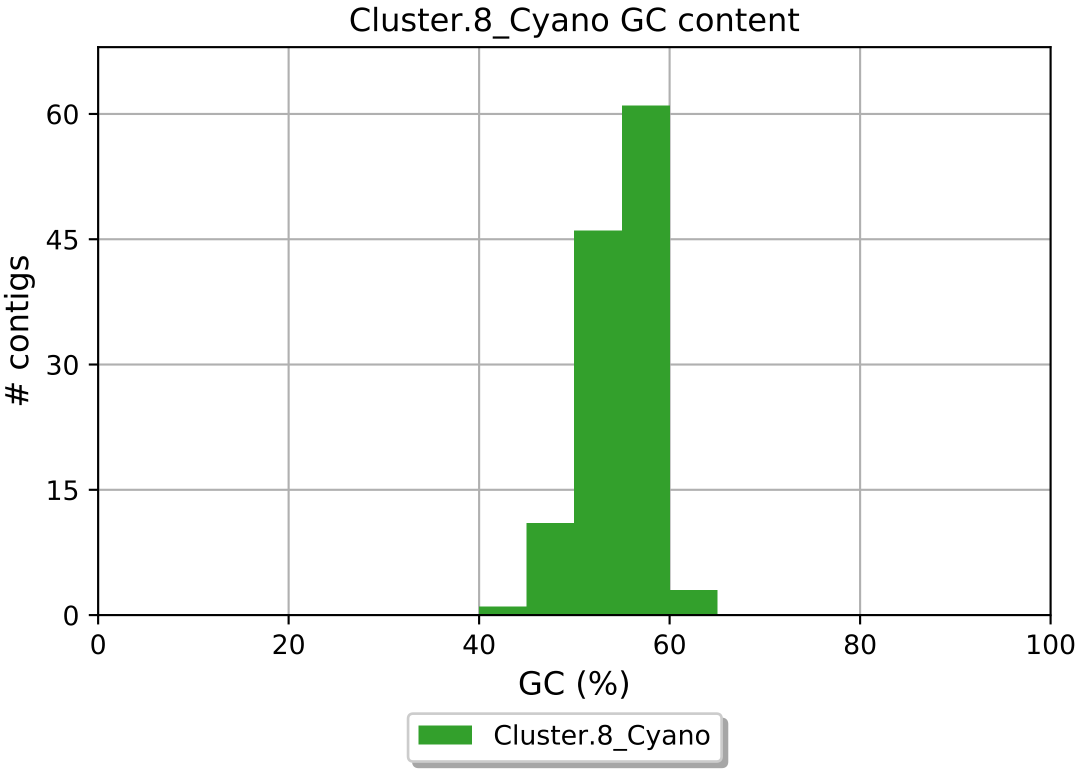
Cluster.8_Cyano GC content
contigs
0
15
30
45
60
0
20
40
60
80
100
GC (%)
Cluster.8_Cyano
