# Supplementary figures and images for: Insights Into Limnothrix sp. Metabolism Based on Comparative Genomics
Source: Front Microbiol. 2018 Nov 20;9:2811. doi: 10.3389/fmicb.2018.02811 (PMC6256058; doi:10.3389/fmicb.2018.02811)

bin.001\_Cyano GC content

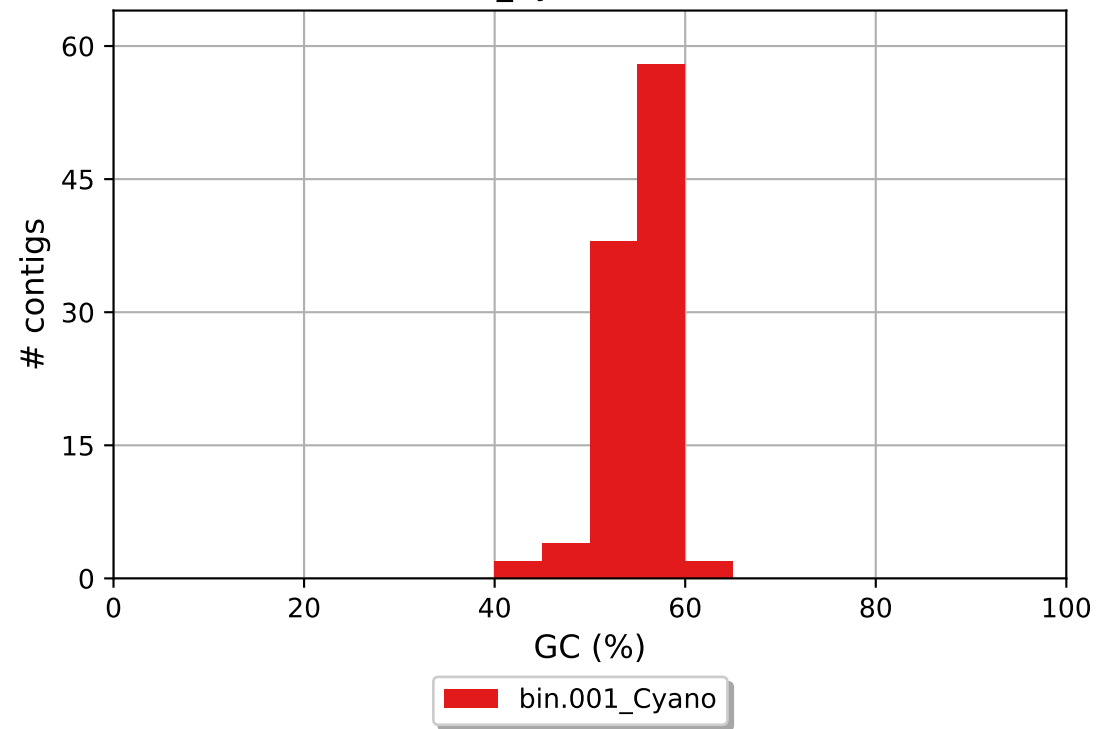

Supplement: Supplementary file 1 [file Data_Sheet_1.ZIP › Newbler_comparisons/quast_results/results_2018_08_03_15_28_44/basic_stats/bin.001_Cyano_GC_content_plot.pdf]

bin.5\_Cyano GC content

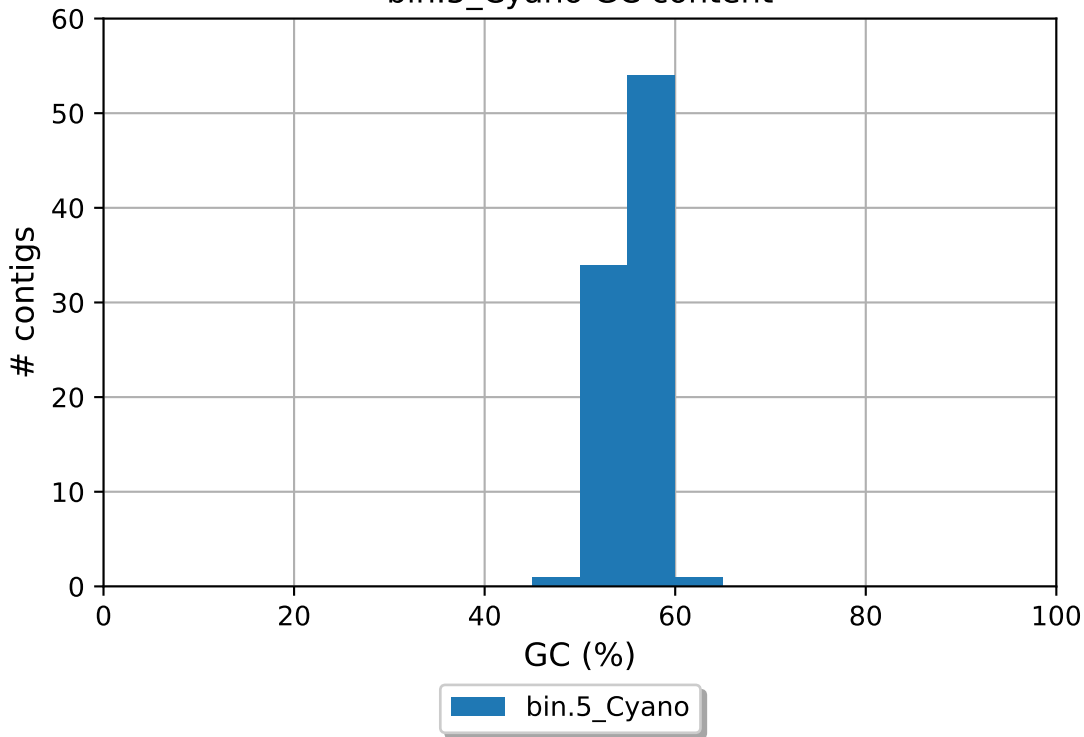

Supplement: Supplementary file 1 [file Data_Sheet_1.ZIP › Newbler_comparisons/quast_results/results_2018_08_03_15_28_44/basic_stats/bin.5_Cyano_GC_content_plot.pdf]

Cluster.8\_Cyano GC content

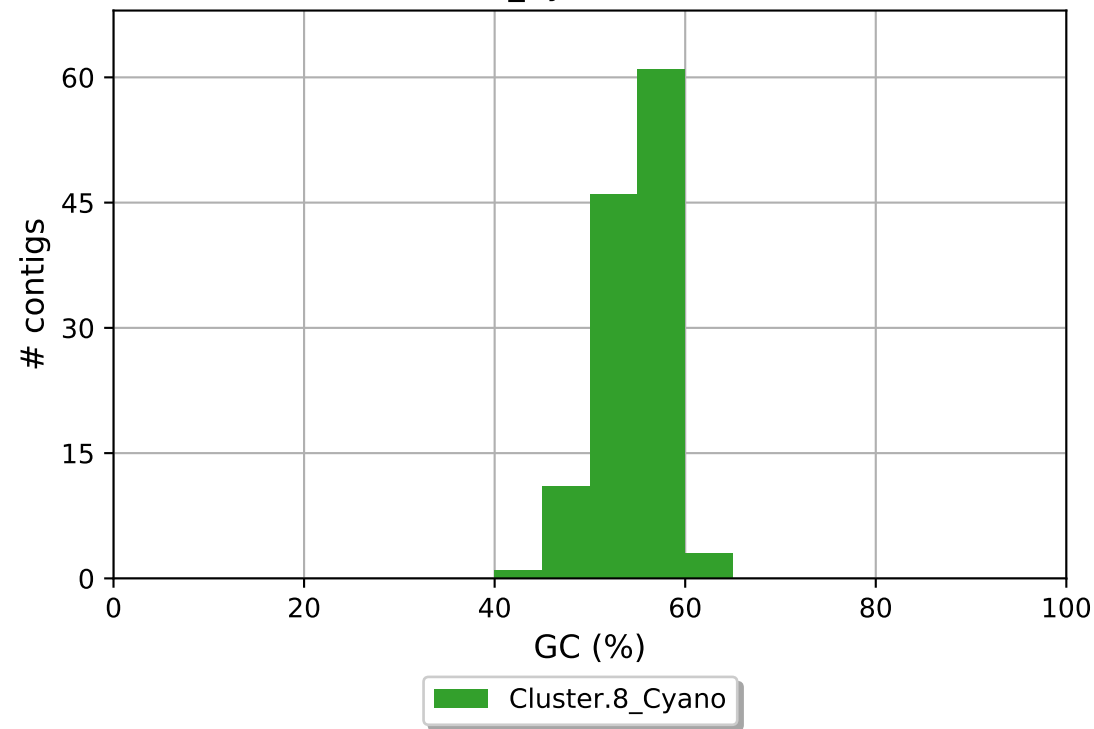

Supplement: Supplementary file 1 [file Data_Sheet_1.ZIP › Newbler_comparisons/quast_results/results_2018_08_03_15_28_44/basic_stats/Cluster.8_Cyano_GC_content_plot.pdf]

Cumulative length

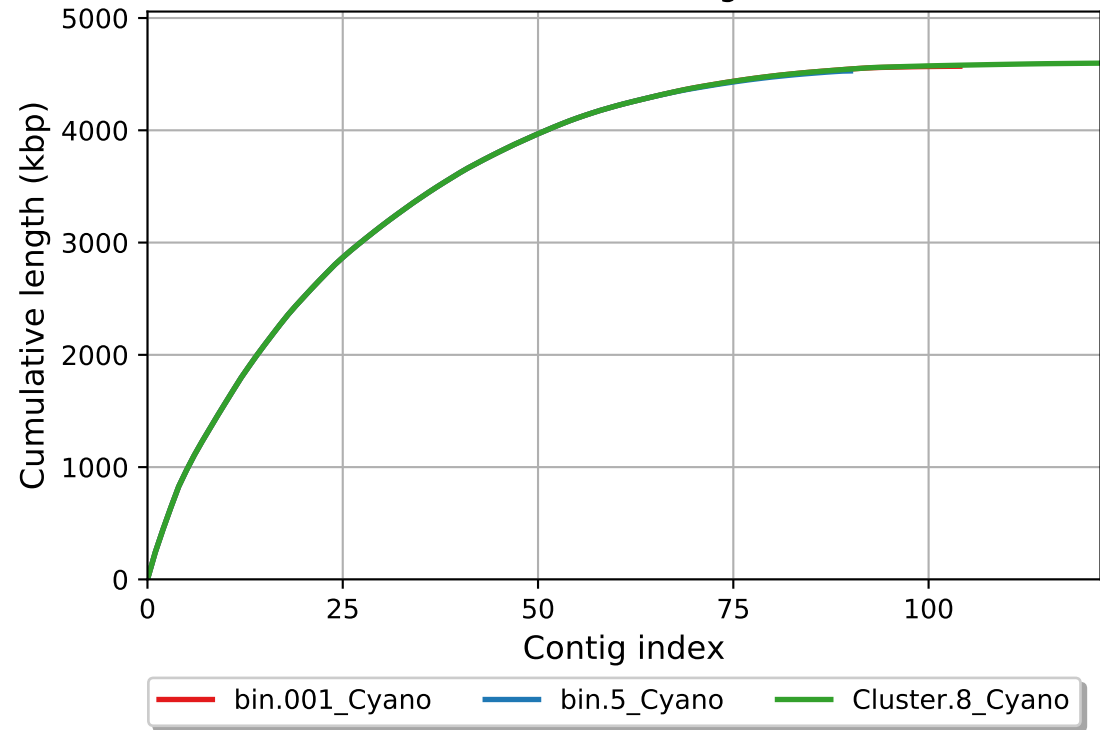

Supplement: Supplementary file 1 [file Data_Sheet_1.ZIP › Newbler_comparisons/quast_results/results_2018_08_03_15_28_44/basic_stats/cumulative_plot.pdf]

# GC content

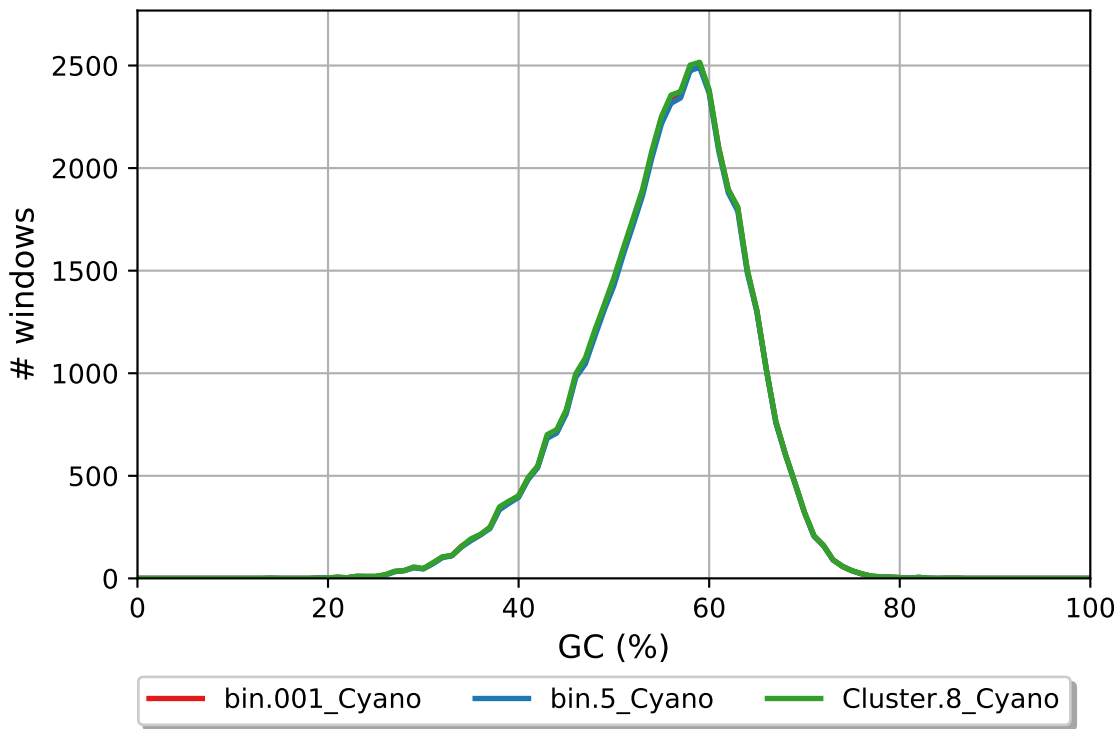

Supplement: Supplementary file 1 [file Data_Sheet_1.ZIP › Newbler_comparisons/quast_results/results_2018_08_03_15_28_44/basic_stats/GC_content_plot.pdf]

Nx

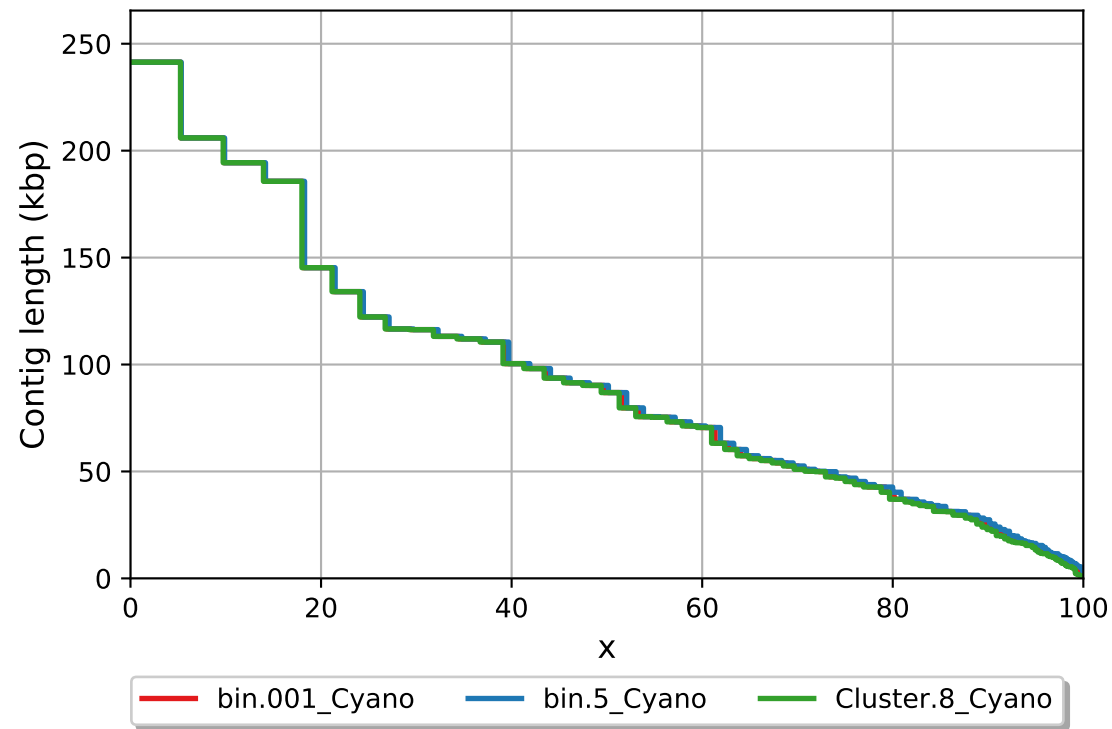

Supplement: Supplementary file 1 [file Data_Sheet_1.ZIP › Newbler_comparisons/quast_results/results_2018_08_03_15_28_44/basic_stats/Nx_plot.pdf]

bin.001\_Cyano coverage histogram (bin size: 1x)

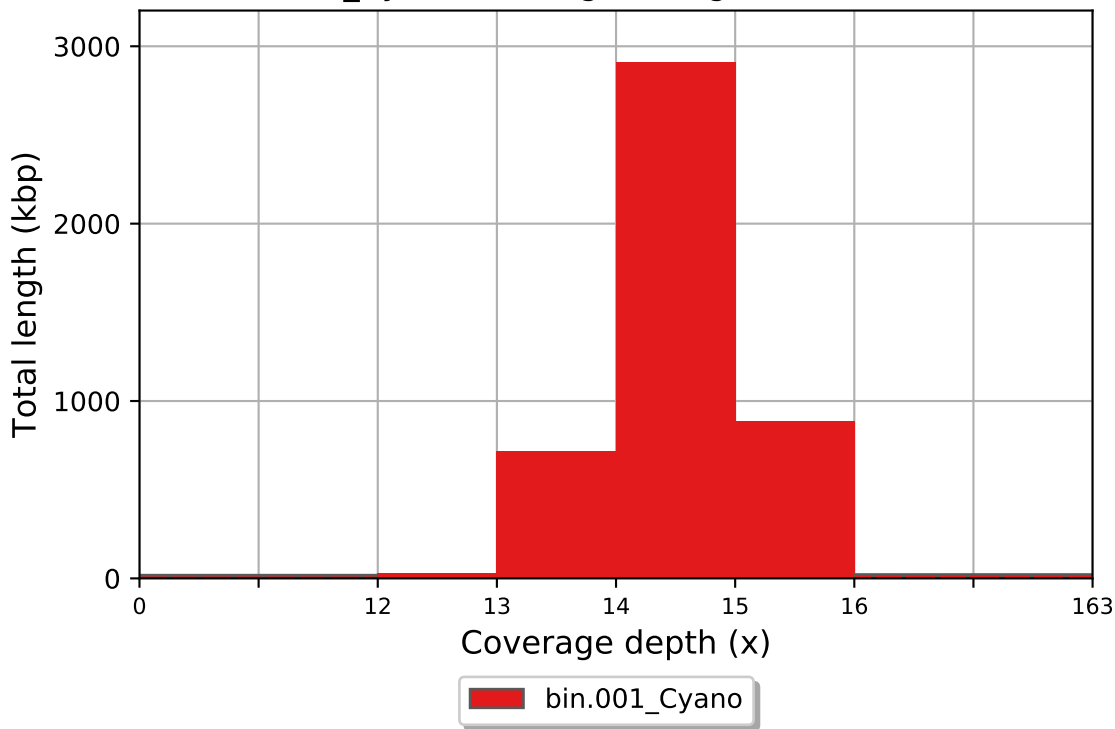

Supplement: Supplementary file 2 [file Data_Sheet_2.ZIP › SPAdes_comparisons/quast_results/results_2018_08_03_15_48_03/basic_stats/bin-001_Cyano_coverage_histogram.pdf]

bin.7\_Cyano coverage histogram (bin size: 1x)

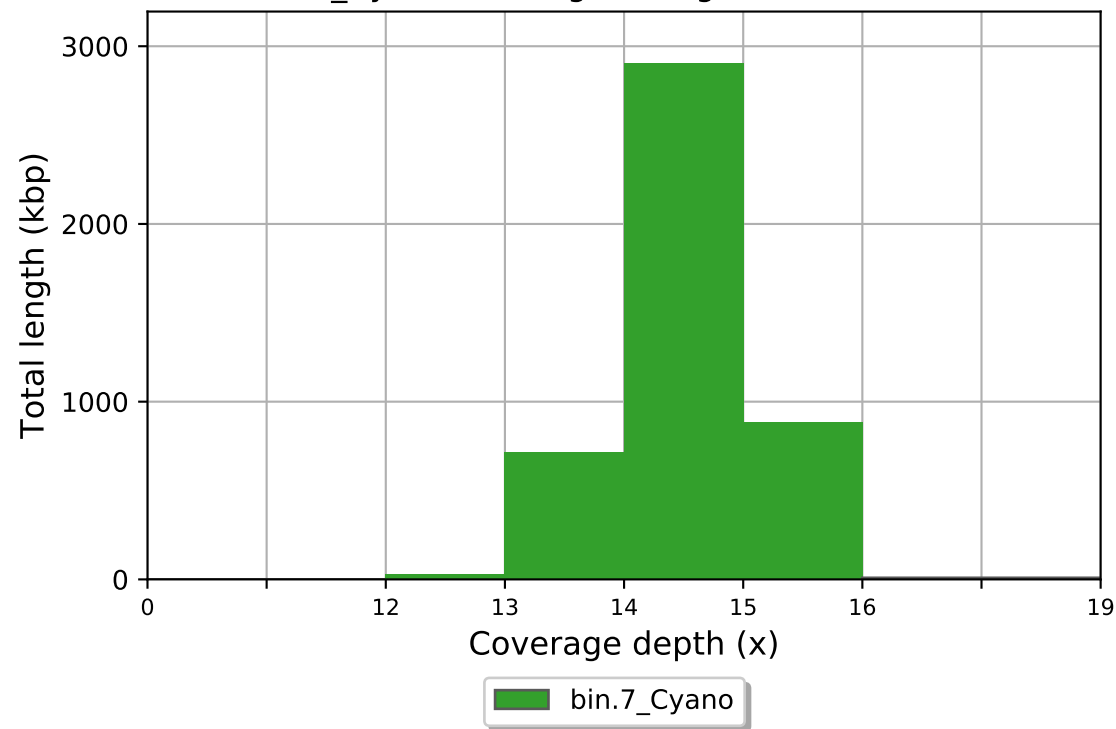

Supplement: Supplementary file 2 [file Data_Sheet_2.ZIP › SPAdes_comparisons/quast_results/results_2018_08_03_15_48_03/basic_stats/bin-7_Cyano_coverage_histogram.pdf]

bin.001\_Cyano GC content

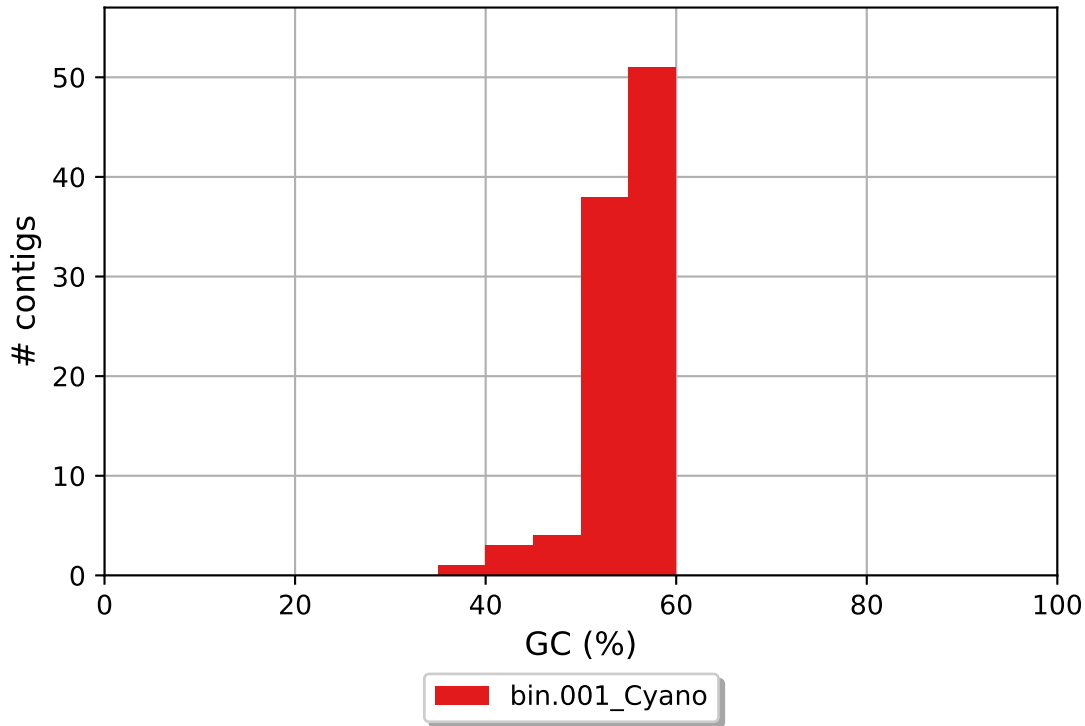

Supplement: Supplementary file 2 [file Data_Sheet_2.ZIP › SPAdes_comparisons/quast_results/results_2018_08_03_15_48_03/basic_stats/bin.001_Cyano_GC_content_plot.pdf]

bin.7\_Cyano GC content

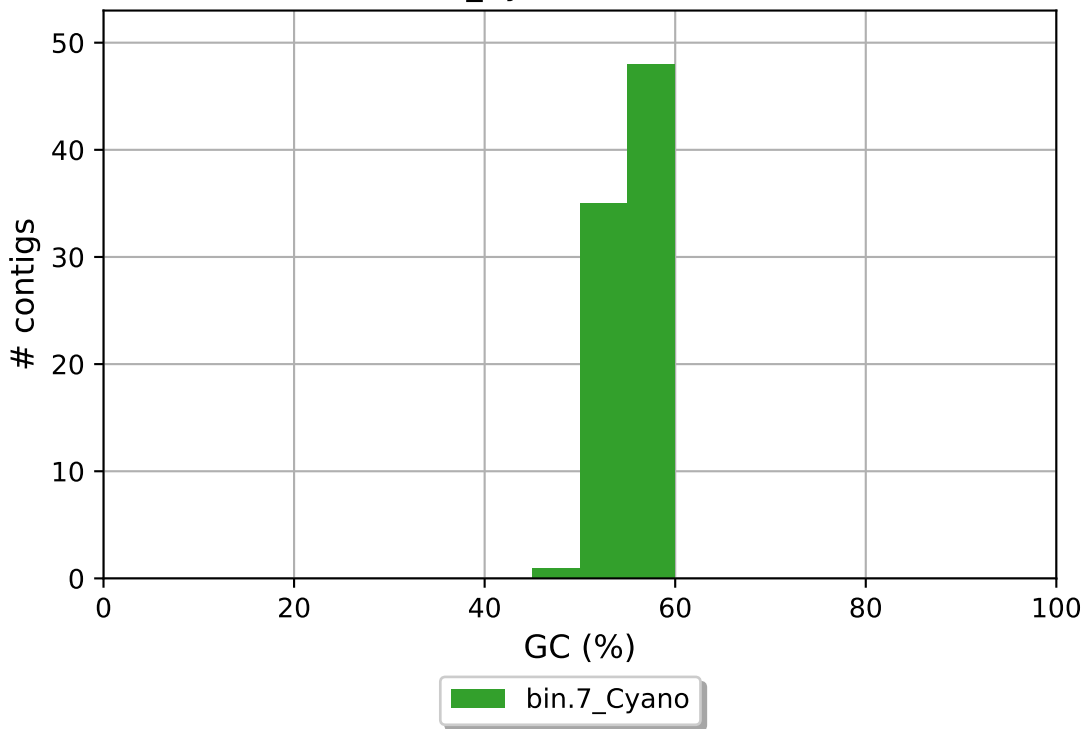

Supplement: Supplementary file 2 [file Data_Sheet_2.ZIP › SPAdes_comparisons/quast_results/results_2018_08_03_15_48_03/basic_stats/bin.7_Cyano_GC_content_plot.pdf]

Cluster.9\_Cyano coverage histogram (bin size: 1x)

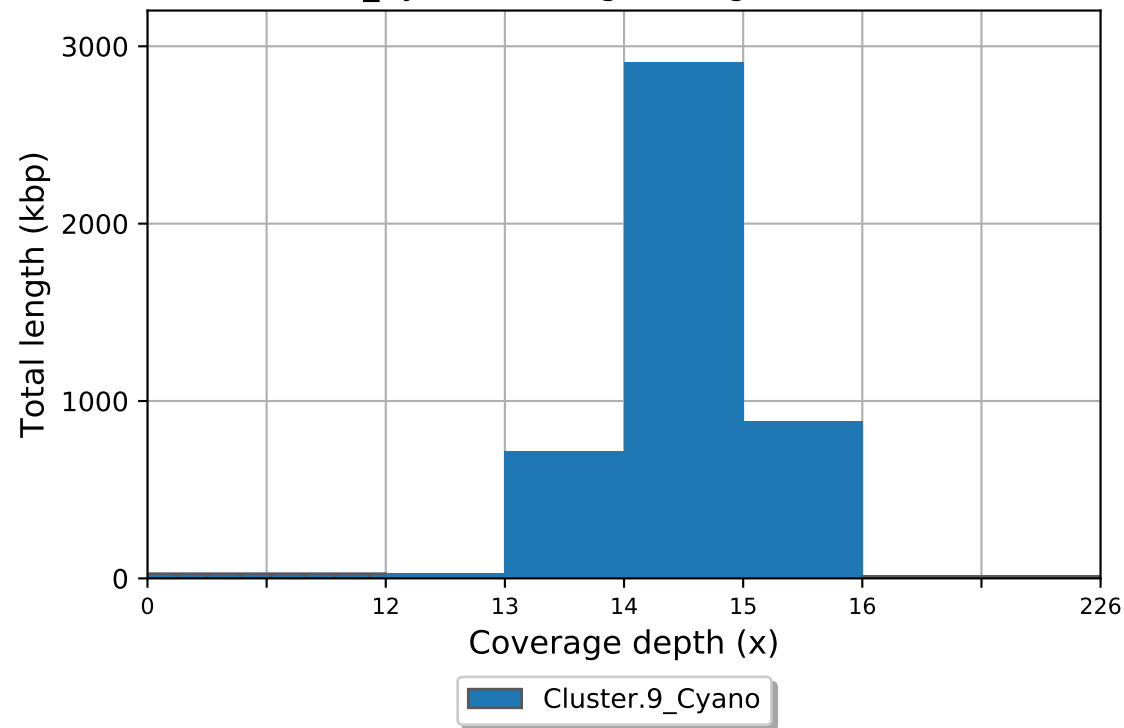

Supplement: Supplementary file 2 [file Data_Sheet_2.ZIP › SPAdes_comparisons/quast_results/results_2018_08_03_15_48_03/basic_stats/Cluster-9_Cyano_coverage_histogram.pdf]

Cluster.9\_Cyano GC content

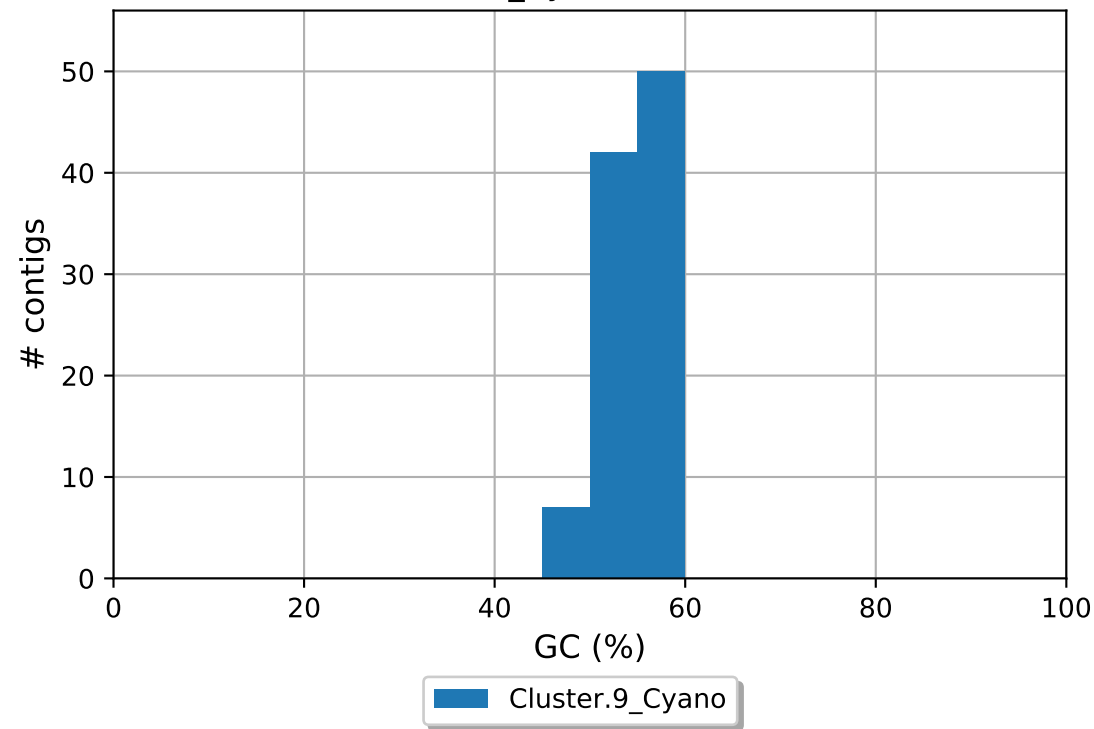

Supplement: Supplementary file 2 [file Data_Sheet_2.ZIP › SPAdes_comparisons/quast_results/results_2018_08_03_15_48_03/basic_stats/Cluster.9_Cyano_GC_content_plot.pdf]

Coverage histogram (bin size: 1x)

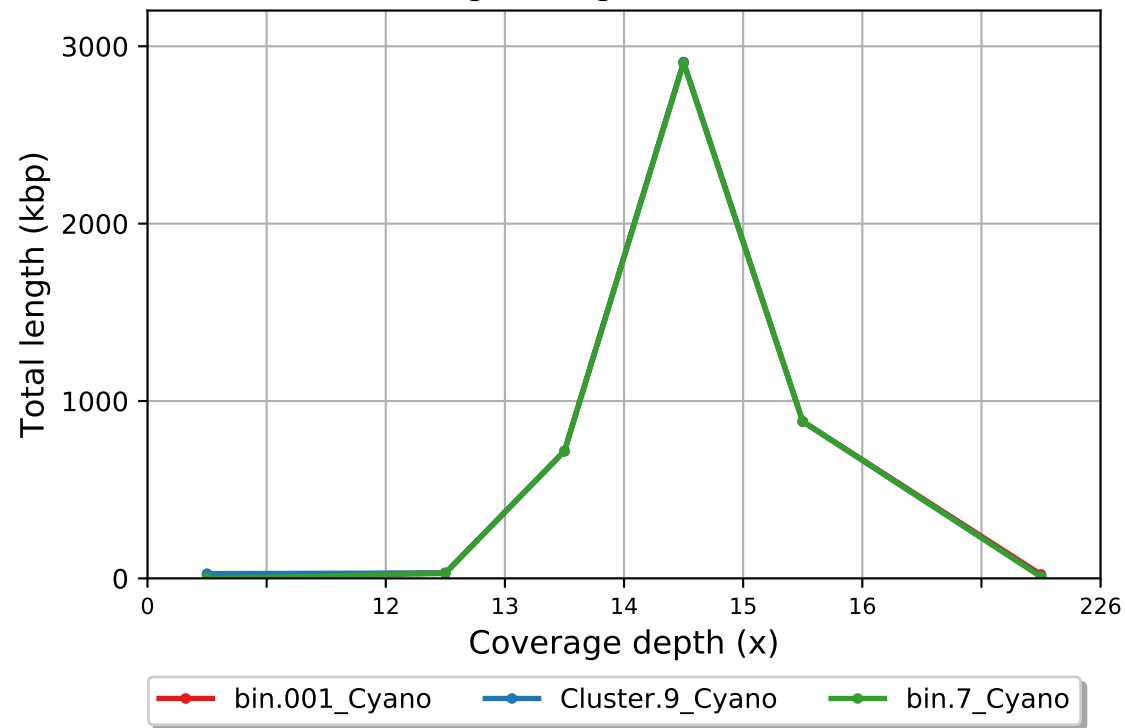

Supplement: Supplementary file 2 [file Data_Sheet_2.ZIP › SPAdes_comparisons/quast_results/results_2018_08_03_15_48_03/basic_stats/coverage_histogram.pdf]

Cumulative length

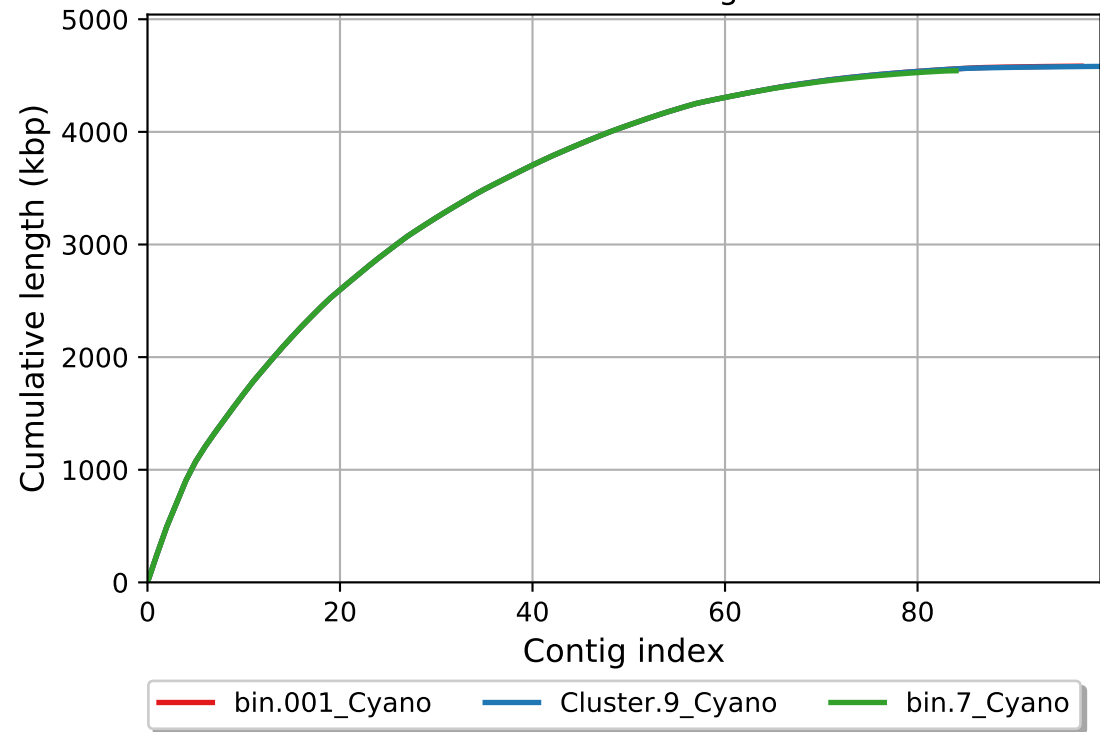

Supplement: Supplementary file 2 [file Data_Sheet_2.ZIP › SPAdes_comparisons/quast_results/results_2018_08_03_15_48_03/basic_stats/cumulative_plot.pdf]

# GC content

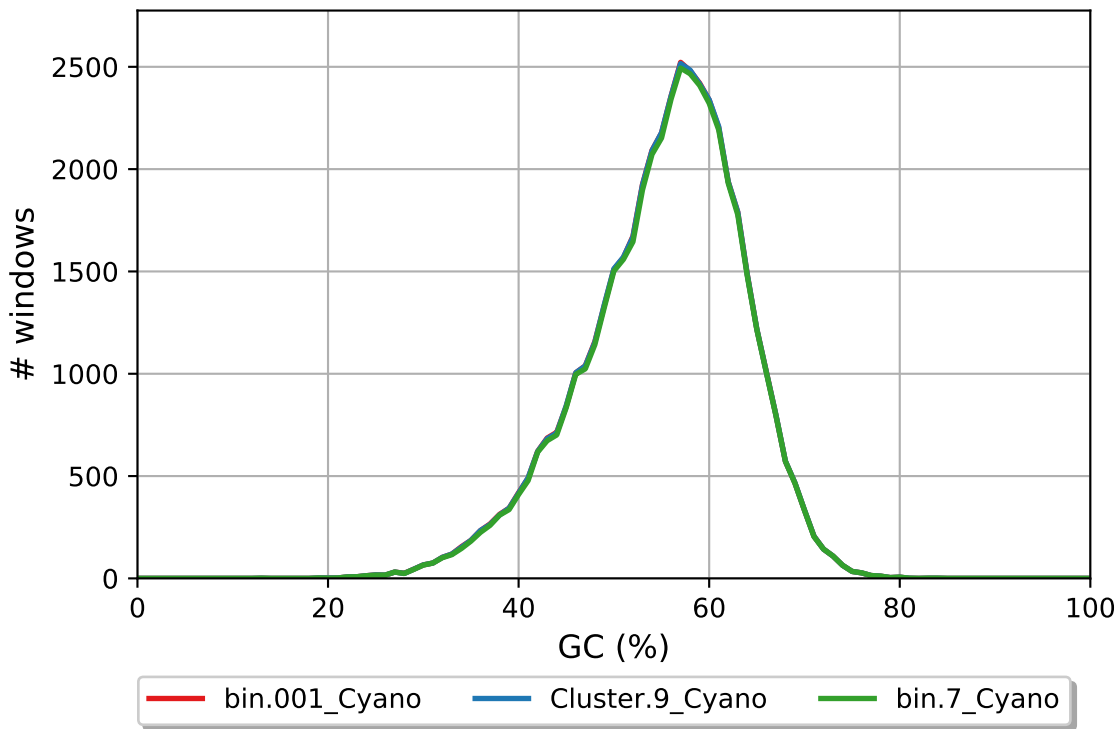

Supplement: Supplementary file 2 [file Data_Sheet_2.ZIP › SPAdes_comparisons/quast_results/results_2018_08_03_15_48_03/basic_stats/GC_content_plot.pdf]

Nx

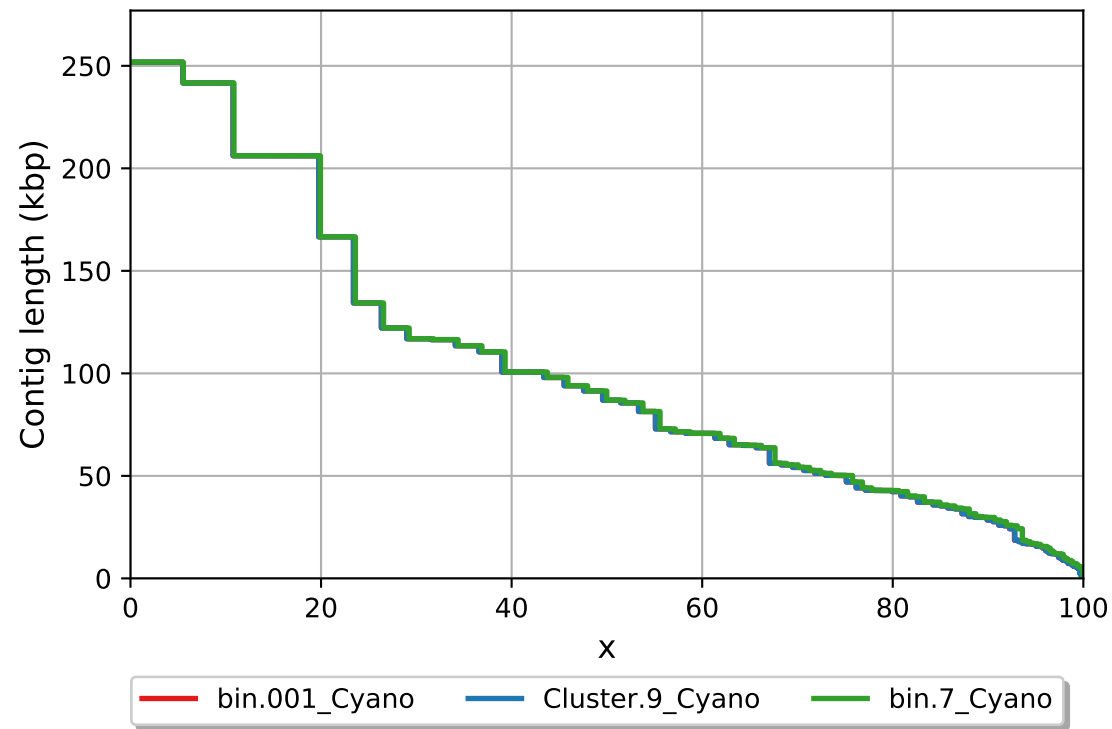

Supplement: Supplementary file 2 [file Data_Sheet_2.ZIP › SPAdes_comparisons/quast_results/results_2018_08_03_15_48_03/basic_stats/Nx_plot.pdf]
