# Supplementary material for: Insights Into Limnothrix sp. Metabolism Based on Comparative Genomics
Source: Front Microbiol. 2018 Nov 20;9:2811. doi: 10.3389/fmicb.2018.02811 (PMC6256058; doi:10.3389/fmicb.2018.02811)
Supplement: Supplementary file 2 [file Data_Sheet_2.ZIP › SPAdes_comparisons/quast_results/results_2018_08_03_15_48_03/icarus.html]

|  |
| --- |
| Icarus **QUAST Contig Browser** by CAB |

**Assemblies:** bin.001\_Cyano, Cluster.9\_Cyano, bin.7\_Cyano| Contig size viewer |
| QUAST report |
