# Supplementary material for: Insights Into Limnothrix sp. Metabolism Based on Comparative Genomics
Source: Front Microbiol. 2018 Nov 20;9:2811. doi: 10.3389/fmicb.2018.02811 (PMC6256058; doi:10.3389/fmicb.2018.02811)
Supplement: Supplementary file 2 [file Data_Sheet_2.ZIP › SPAdes_comparisons/quast_results/results_2018_08_03_15_48_03/report.pdf]

Report

	bin.001_Cyano	Cluster.9_Cyano	bin.7_Cyano
# contigs (>= 0 bp)	97	99	84
# contigs (>= 1000 bp)	93	93	84
# contigs (>= 5000 bp)	84	83	81
# contigs (>= 10000 bp)	72	72	71
# contigs (>= 25000 bp)	56	56	56
# contigs (>= 50000 bp)	34	34	34
Total length (>= 0 bp)	4583395	4581382	4542673
Total length (>= 1000 bp)	4580208	4576189	4542673
Total length (>= 5000 bp)	4559535	4554247	4531738
Total length (>= 10000 bp)	4473906	4473906	4457098
Total length (>= 25000 bp)	4227867	4227867	4227867
Total length (>= 50000 bp)	3442792	3442792	3442792
# contigs	97	99	84
Largest contig	251784	251784	251784
Total length	4583395	4581382	4542673
GC (%)	55.20	55.21	55.24
N50	86917	86917	86917
N75	50181	50181	50181
L50	17	17	17
L75	34	34	34
# N's per 100 kbp	0.00	0.00	0.00

All statistics are based on contigs of size >= 500 bp, unless otherwise noted
(e.g., "# contigs (>= 0 bp)" and "Total length (>= 0 bp)" include all contigs).

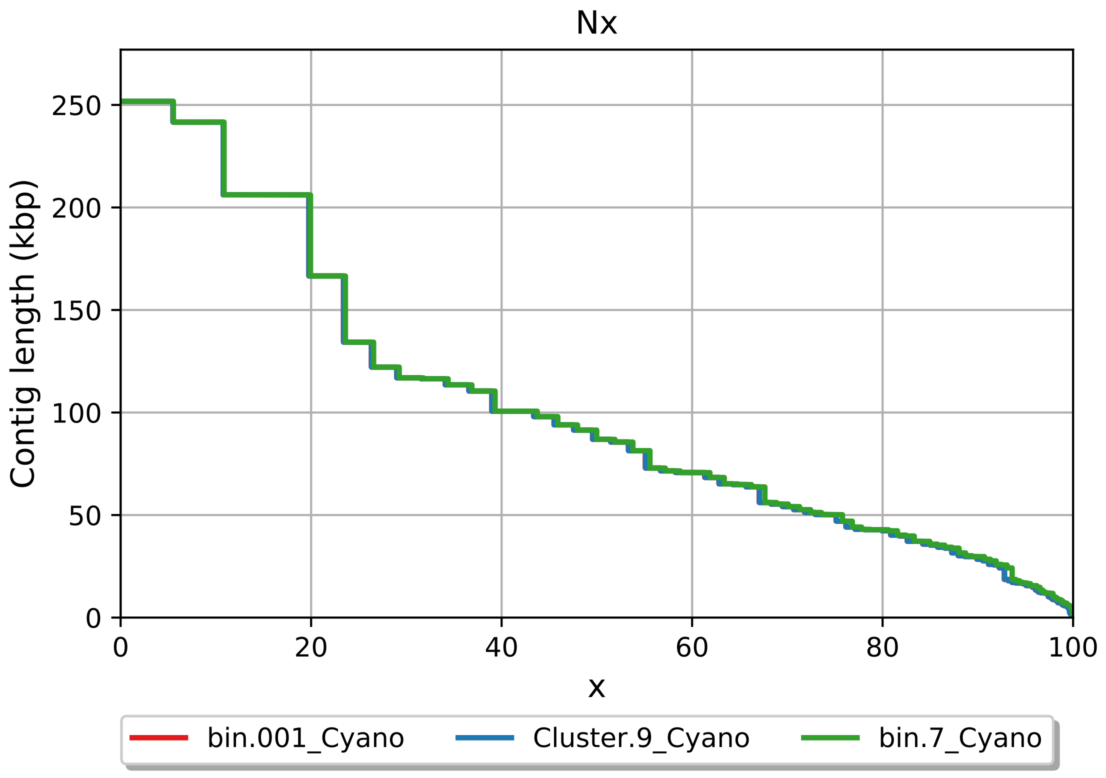

Nx
Contig length (kbp)
0
50
100
150
200
250
0
20
40
60
80
100
x
bin.001_Cyano
Cluster.9_Cyano
bin.7_Cyano

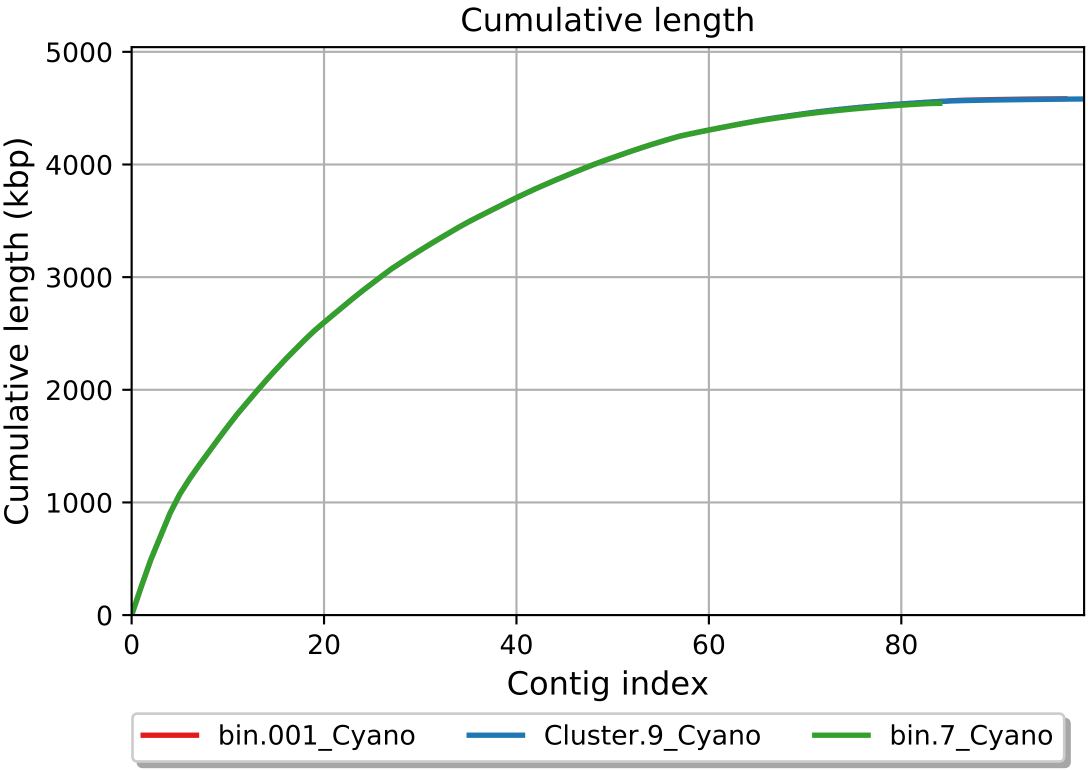

Cumulative length
Cumulative length (kbp)
5000
4000
3000
2000
1000
0
0
20
40
60
80
Contig index
bin.001_Cyano
Cluster.9_Cyano
bin.7_Cyano

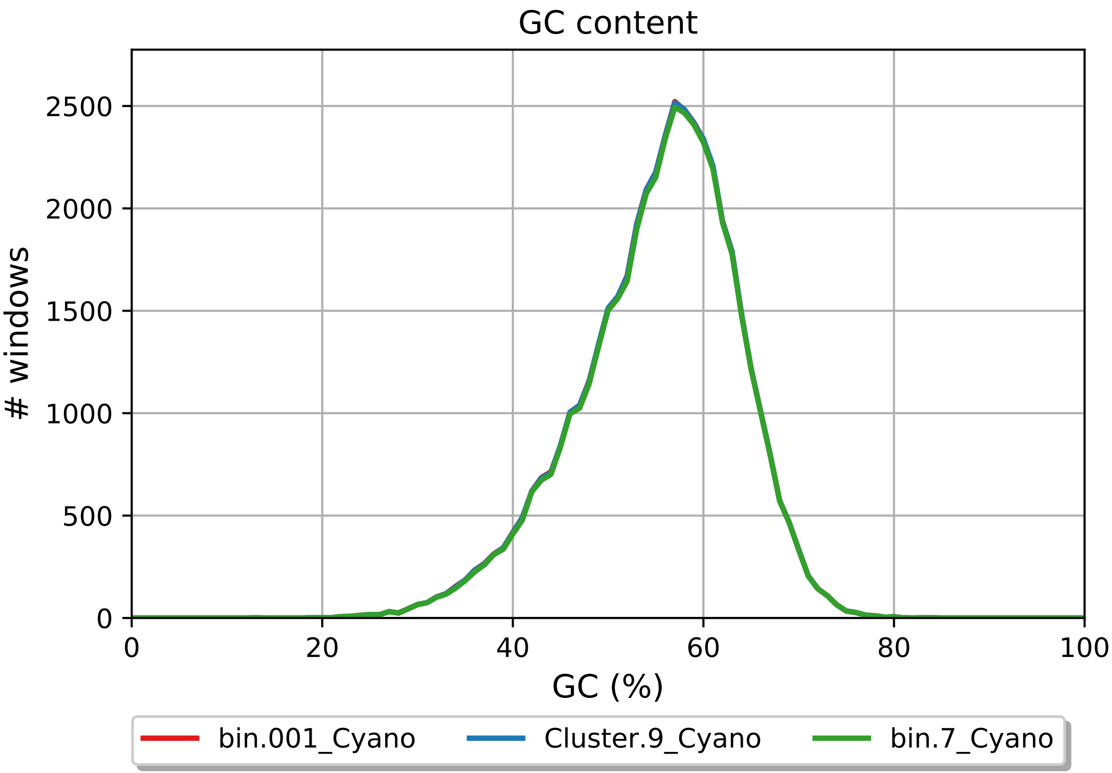

GC content
windows
2500
2000
1500
1000
500
0
0
20
40
60
80
100
GC (%)
bin.001_Cyano
Cluster.9_Cyano
bin.7_Cyano

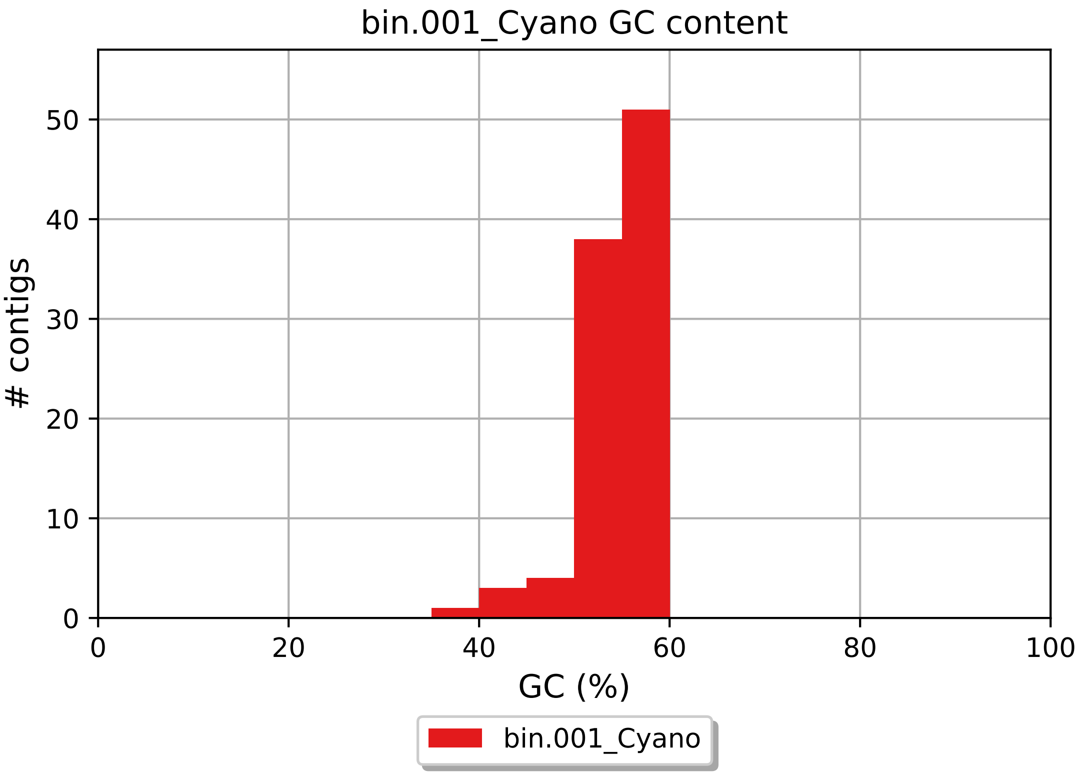

bin.001_Cyano GC content
contigs
0
10
20
30
40
50
0
20
40
60
80
100
GC (%)
bin.001_Cyano

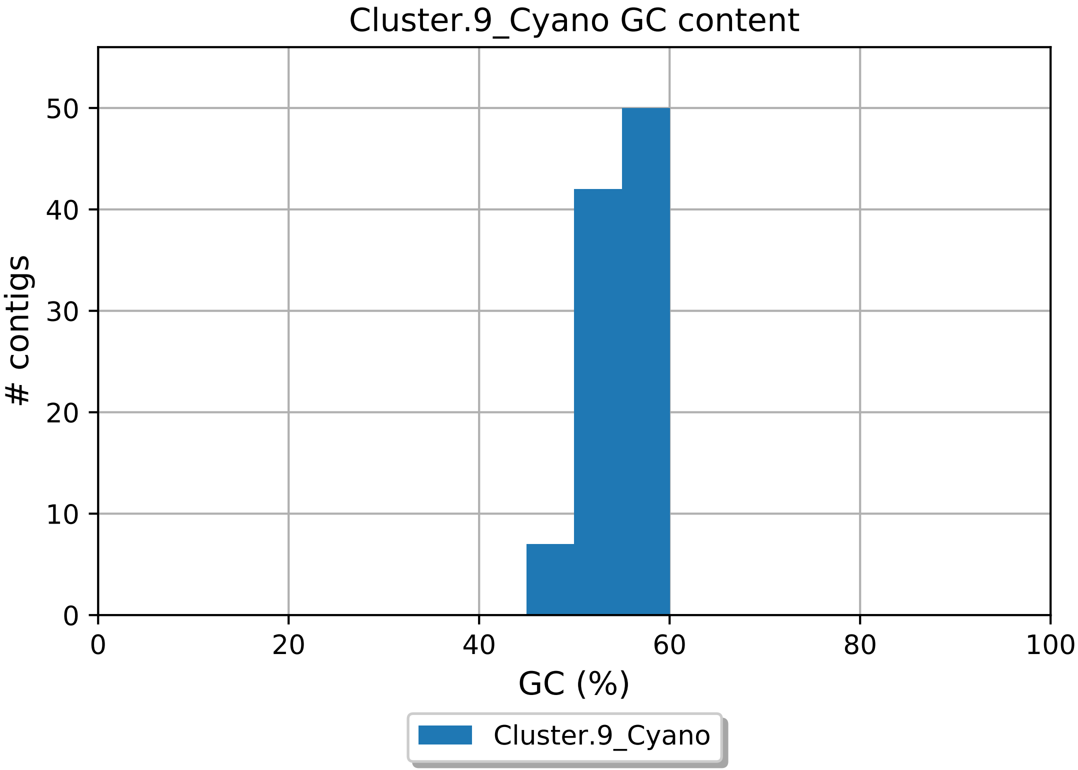

Cluster.9_Cyano GC content
contigs
0
10
20
30
40
50
0
20
40
60
80
100
GC (%)
Cluster.9_Cyano

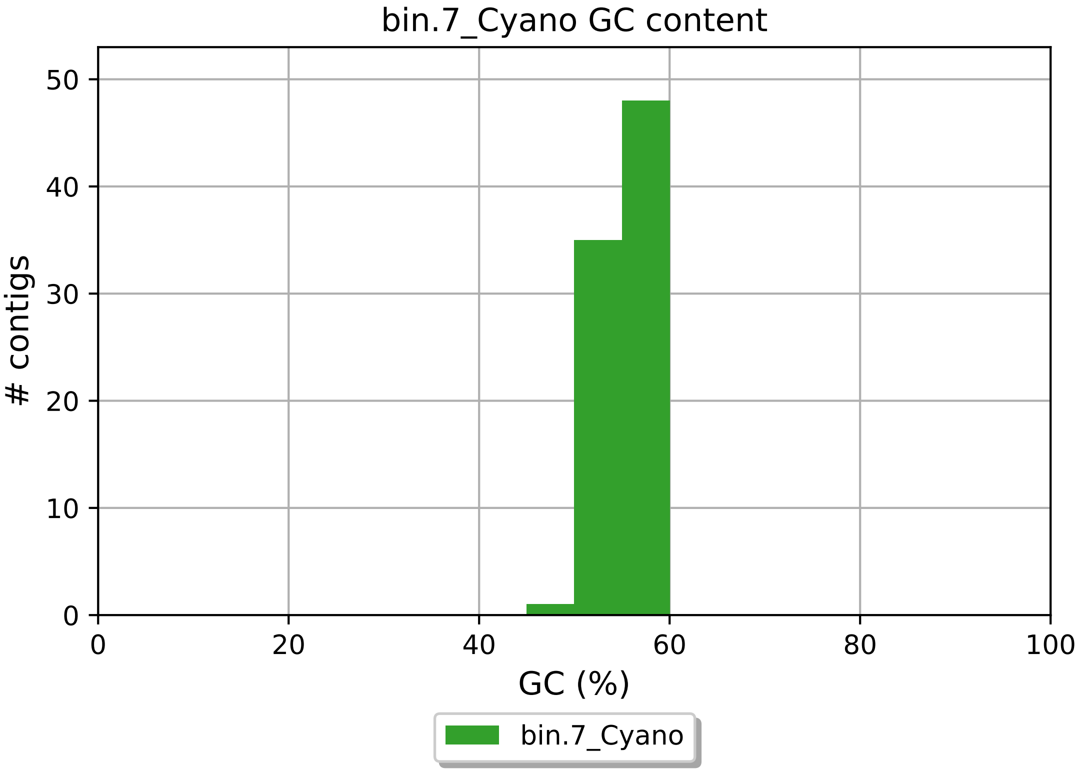

bin.7_Cyano GC content
contigs
0
10
20
30
40
50
0
20
40
60
80
100
GC (%)
bin.7_Cyano

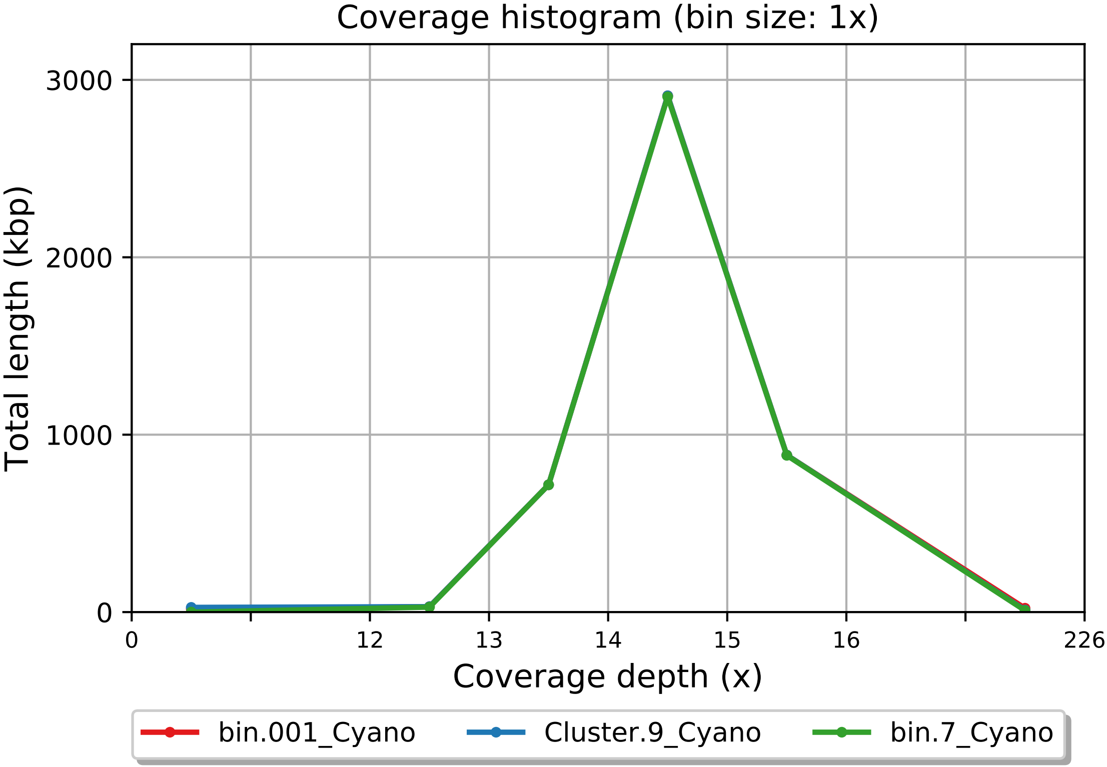

Coverage histogram (bin size: 1x)
Total length (kbp)
3000
2000
1000
0
0
12
13
14
15
16
226
Coverage depth (x)
bin.001_Cyano
Cluster.9_Cyano
bin.7_Cyano

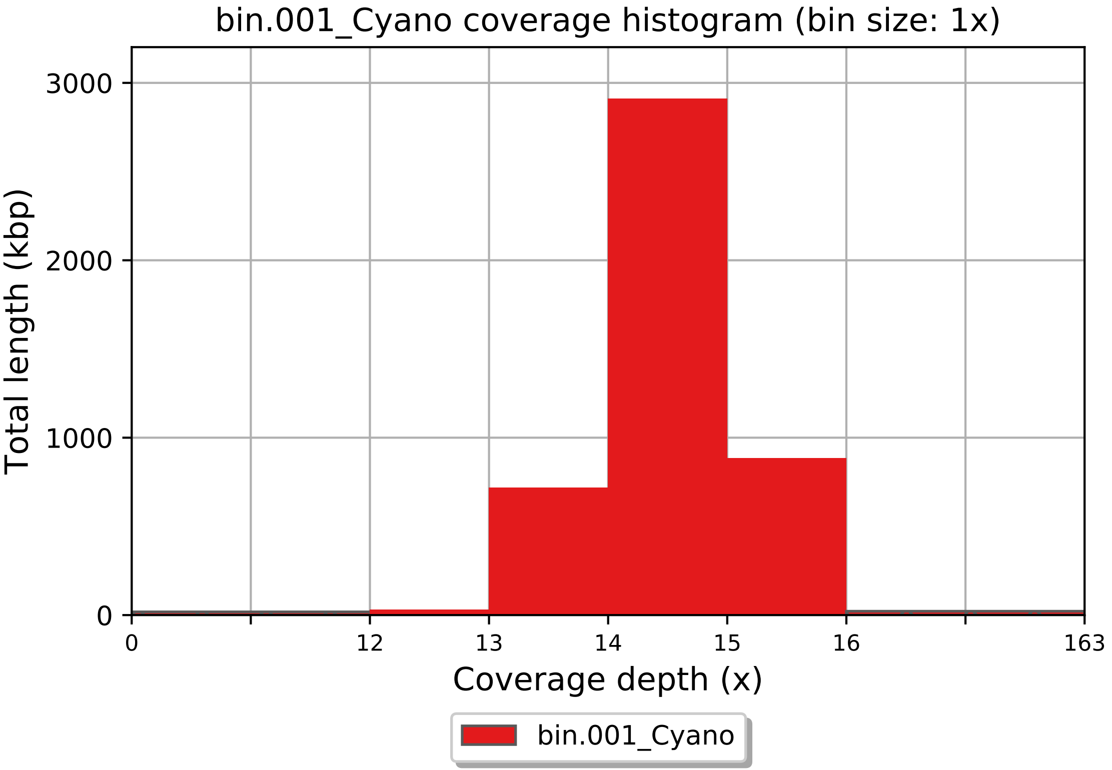

bin.001_Cyano coverage histogram (bin size: 1x)
Total length (kbp)
3000
2000
1000
0
0
12
13
14
15
16
163
Coverage depth (x)
bin.001_Cyano

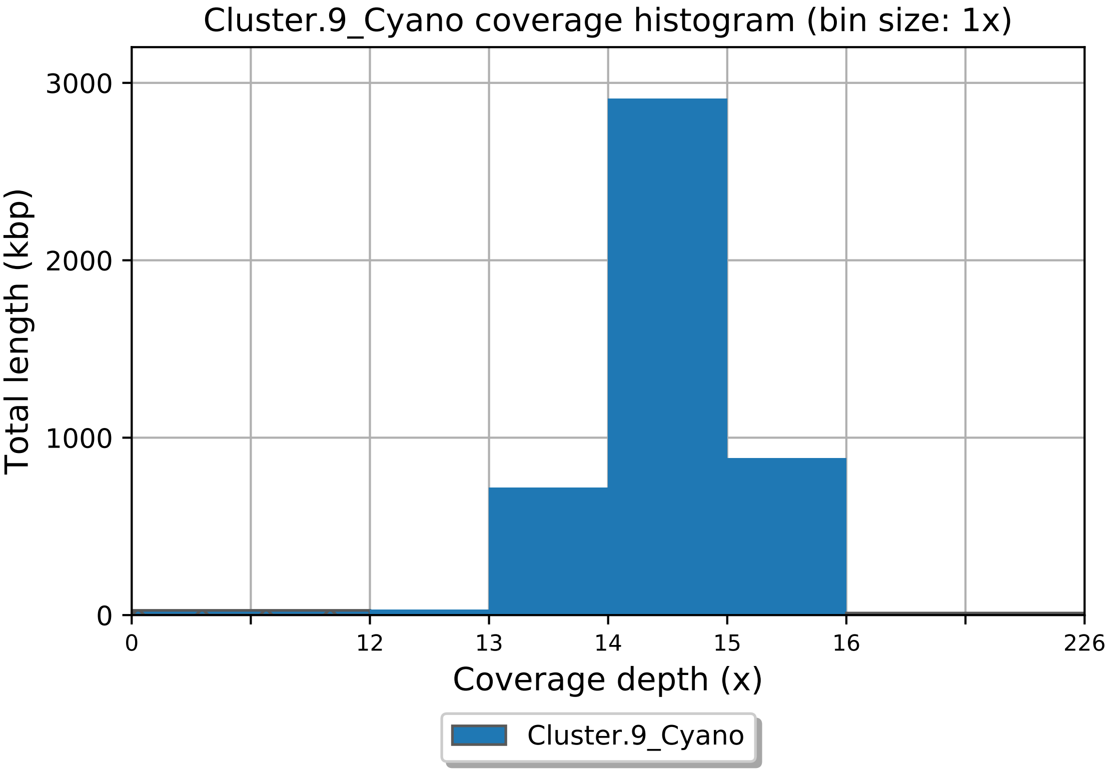

Cluster.9_Cyano coverage histogram (bin size: 1x)
Total length (kbp)
3000
2000
1000
0
0
12
13
14
15
16
226
Coverage depth (x)
Cluster.9_Cyano

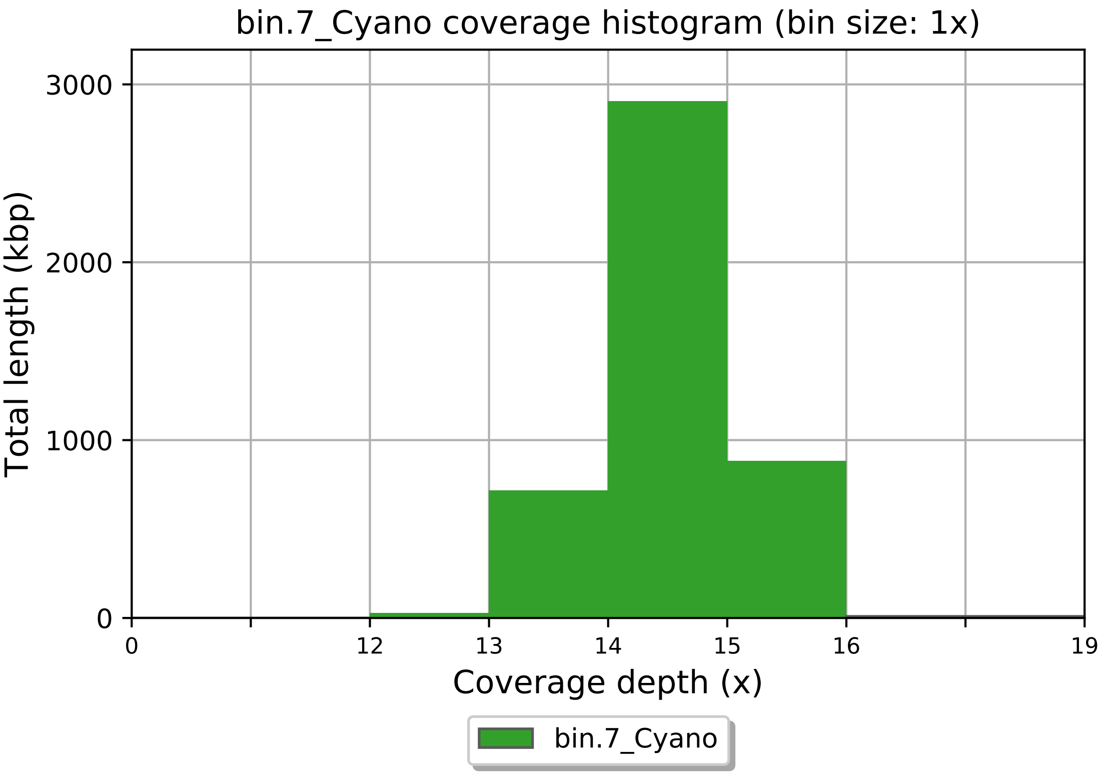
bin.7_Cyano coverage histogram (bin size: 1x)
Total length (kbp)
3000
2000
1000
0
0
12
13
14
15
16
19
Coverage depth (x)
bin.7_Cyano
