# Supplementary material for: Insights Into Limnothrix sp. Metabolism Based on Comparative Genomics
Source: Front Microbiol. 2018 Nov 20;9:2811. doi: 10.3389/fmicb.2018.02811 (PMC6256058; doi:10.3389/fmicb.2018.02811)
Supplement: Supplementary file 4 [file Data_Sheet_4.PDF]

Supplementary Material

Insights Into *Limnothrix* sp. Metabolism Based On Comparative Genomics of the Amazonian CACIAM 69d Strain

Alex Ranieri Jerônimo Lima*, Andrei Santos Siqueira, Janaina Mota de Vasconcelos, James Siqueira Pereira, Juliana Simão Nina de Azevedo, Pablo Henrique Gonçalves Moraes, Délia Cristina Figueira Aguiar, Clayton Pereira Silva de Lima, João Lídio Silva Gonçalves Vianez-Júnior, Márcio Roberto Teixeira Nunes, Luciana Pereira Xavier, Leonardo Teixeira Dall´Agnol & Evonnildo Costa Goncalves

*** Correspondence:** Alex Ranieri Jerônimo Lima: alex.lima@icb.ufpa.br, alexranieri@hotmail.com

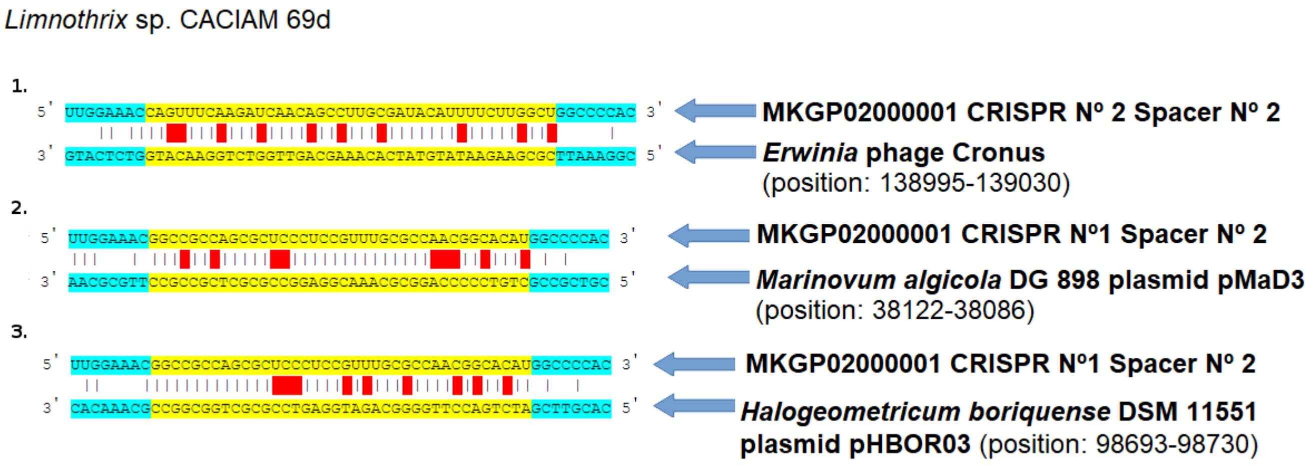

Supplementary Figure 1. Alignments of *Limnothrix* sp. CACIAM 69d CRISPR spacers and targets (protospacers), predicted by CRISPRTarget.

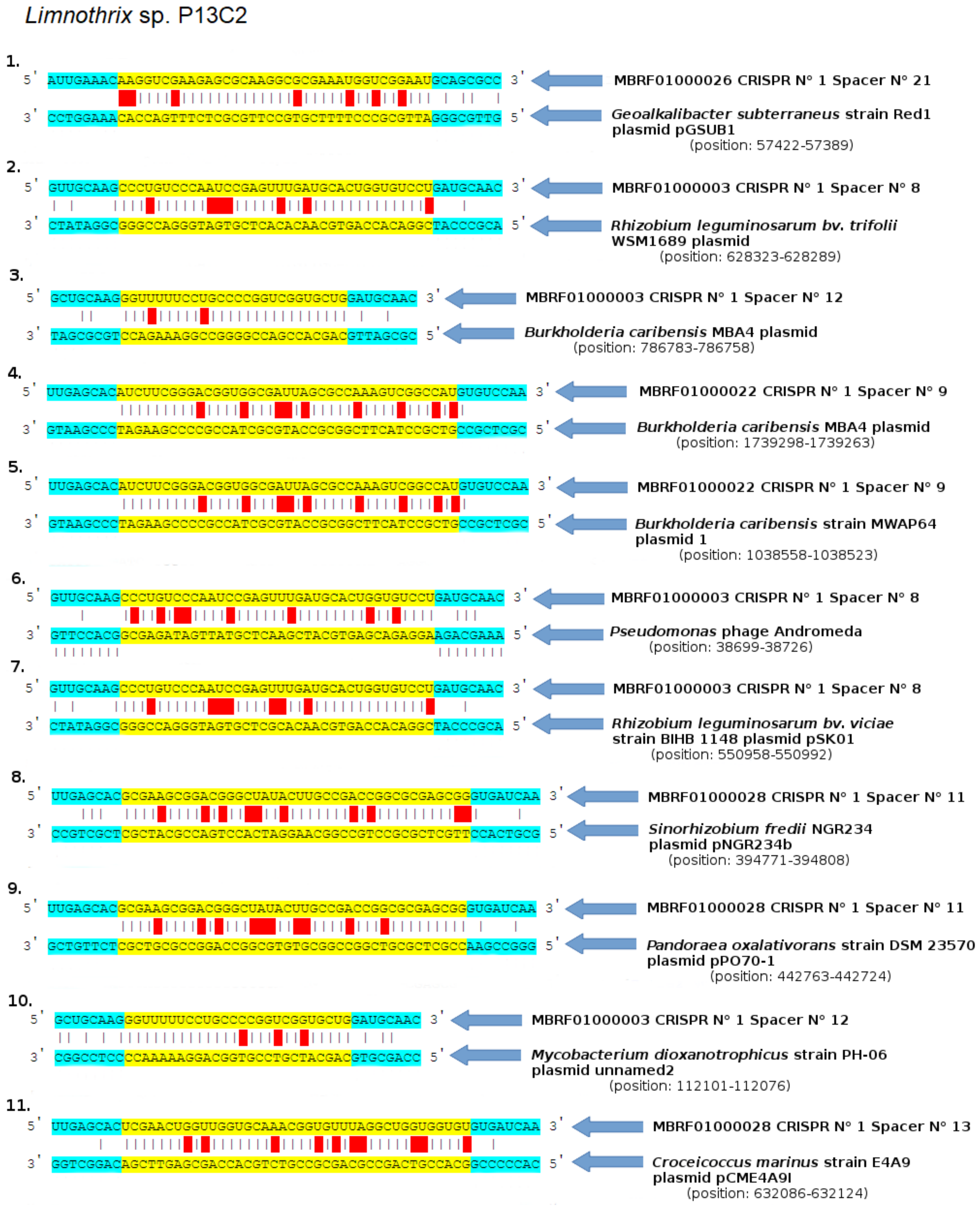

Supplementary Figure 2. Alignments of *Limnothrix* sp. P13C2 CRISPR spacers and targets (protospacers), predicted by CRISPRTarget.

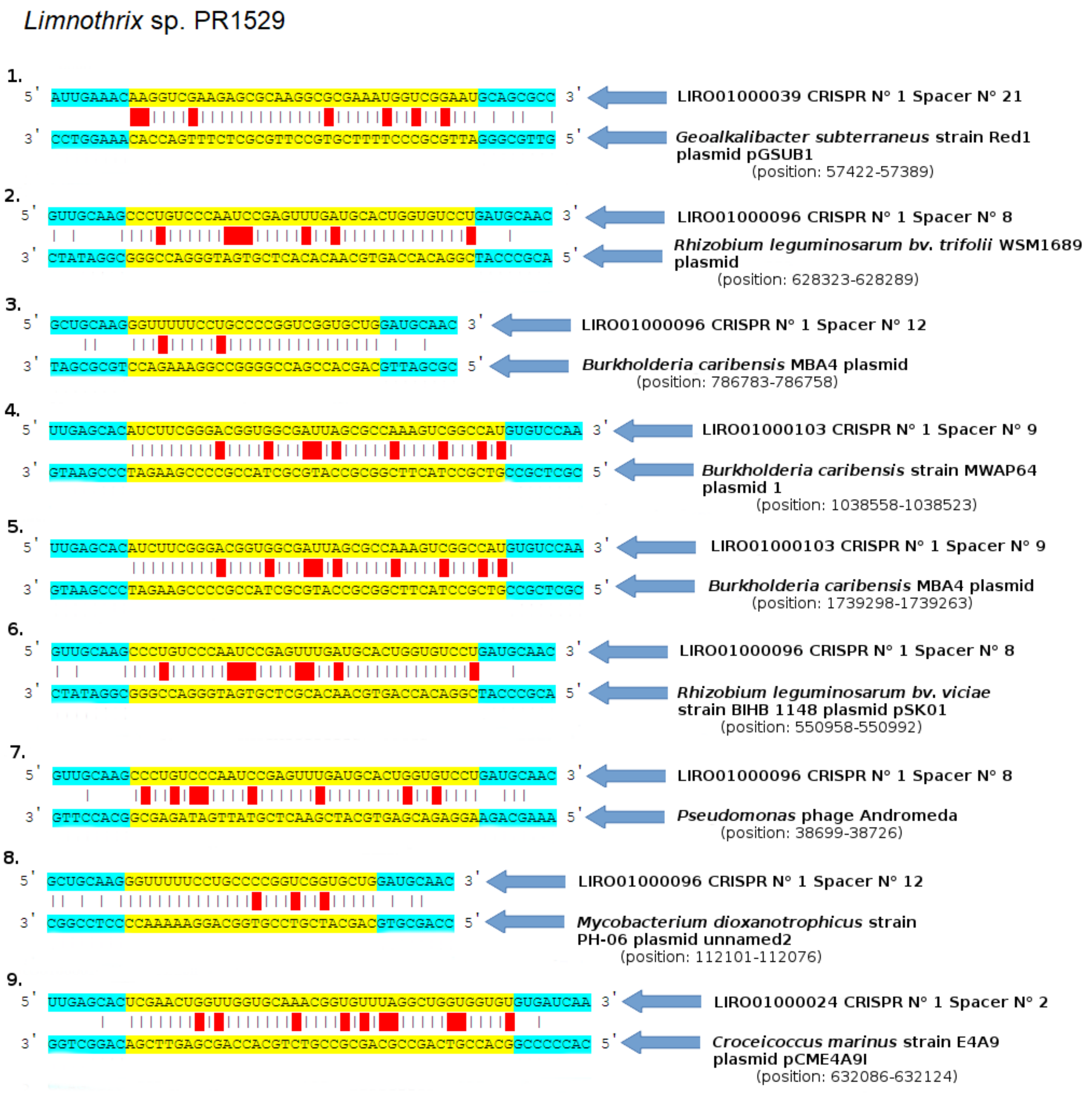

Supplementary Figure 3. Alignments of *Limnothrix* sp. PR1529 CRISPR spacers and targets (protospacers), predicted by CRISPRTarget.

Limnothrix rosea IAM M-220 NIES-208

1.

5' ACUGAAAGUCUCACCAUAAUCAAAAAUUGUGCCAUCAAAAUAAUUCUCUCAAG 3' — MRBY01000050 CRISPR N° 1 Spacer N° 63

3' TTAGTATAAGAGTGGAAATGGTTTTTAGGACGGTATTTCTATTAAAAGTTAAT 5' — ***Bacillus thuringiensis*** **Bt18247 plasmid p81952**
(position: 289-325)

2.

5' ACUGAAAGUCUCACCAUAAUCAAAAAUUGUGCCAUCAAAAUAAUUCUCUCAAG 3' — MRBY01000050 CRISPR N° 1 Spacer N° 63

3' TTAGTATAAGAGTGGAAATGGTTTTTAAGACGGTATTTCTATTAGAAGTTAAT 5' — ***Bacillus thuringiensis*** **strain MYBT18246 plasmid p142098**
(position: 82856-82821)

3.

5' ACUGAAACAAUGAAGAAACCAAGGAAAGUUAUGGAAGACGUAAAACUUUCGAU 3' — MRBY01000014 CRISPR N° 1 Spacer N° 78

3' CTTCGCGTTTACTTACTTGGGTACTTTCAATACCTTTGGGACTTTGGACAAAG 5' — ***Rufibacter tibetensis*** **strain 1351 plasmid 1**
(position: 8331-8295)

4.

5' ACUGAAAGAAUGCAUUGUUUUUCGUUGGCAUCAUCAACGCUAACUCUCAAG 3' — MRBY01000050 CRISPR N° 1 Spacer N° 79

3' GATTTCTTTTAGGTAACGTCTAACAACCGTGGTAGTTGCGATTAGTACGCG 5' — ***Escherichia coli*** **K-12 strain DHB4 plasmid F128-(DHB4)**
(position: 204314-204348)

5.

5' ACUGAAAGAAUGCAUUGUUUUUCGUUGGCAUCAUCAACGCUAACUCUCAAG 3' — MRBY01000050 CRISPR N° 1 Spacer N° 79

3' GATTTCTTTTAGGTAACGTCTAACAACCGTGGTAGTTGCGATTAGTACGCG 5' — ***Escherichia coli*** **K-12 strain C3026 plasmid F128-(C3026)**
(position: 185269-185303)

6.

5' ACUGAAACGAGGAGCUAGAAAAAUUUAUUGAUGACCUUGCUAACUUUCGAU 3' — MRBY01000014 CRISPR N° 1 Spacer N° 74

3' TTTTCTTCCTGCTCAATCTTTTGAAATTTTTACTTGAACGATTTATAGGAC 5' — ***Lactobacillus salivarius*** **strain ZLS006 plasmid unnamed1**
(position: 214258-214292)

7.

5' ACUGAAAGGGCAAUAAAAAAAGCAGCCCGAAAGCUGCUAGAGGACUCUCAAG 3' — MRBY01000050 CRISPR N° 1 Spacer N° 35

3' AAAAAATACCTTTATTTTTTCGTCGGGATTCTGACGATTACGTTATTATGT 5' — ***Mannheimia*** **phage vB_MhS_535AP2**
(position: 24318-24289)

8.

5' ACUGAAAGGGCAAUAAAAAAAGCAGCCCGAAAGCUGCUAGAGGACUCUCAAG 3' — MRBY01000050 CRISPR N° 1 Spacer N° 35

3' AAAAAATACCTTTATTTTTTCGTCGGGATTCTGACGATTACGTTATTATGT 5' — ***Mannheimia*** **phage vB_MhS_587AP2**
(position: 23398-23369)

9.

5' ACUGAAAGUUUUUCAAGAUACGUUUAUCGUCGCAUAUUAUUUUCUCUCAAG 3' — MRBY01000050 CRISPR N° 1 Spacer N° 52

3' GGGTCTCGAAAAAGTTCTATGTAGAGATCTCCGAATAATAAAAATTACTGC 5' — ***Acinetobacter haemolyticus*** **strain TJS01 plasmid pAHTJS1**
(position: 18715-18681)

10.

5' ACUGAAAGUUUUUCAAGAUACGUUUAUCGUCGCAUAUUAUUUUCUCUCAAG 3' — MRBY01000050 CRISPR N° 1 Spacer N° 52

3' GAGGAAATAAAAAGTACTATGCAAATAATATAGTATCATAATAATTATCAC 5' — ***Leuconostoc*** **phage CHA**
(position: 11676-11644)

11.

5' ACUGAAAGUUUUUCAAGAUACGUUUAUCGUCGCAUAUUAUUUUCUCUCAAG 3' — MRBY01000050 CRISPR N° 1 Spacer N° 52

3' GAGGAAATAAAAAGTACTATGCAAATAATATAGTATCATAATAATTATCAC 5' — ***Leuconostoc*** **phage phiLNTR3**
(position: 11665-11633)

12.

5' ACUGAAAGUUUUUCAAGAUACGUUUAUCGUCGCAUAUUAUUUUCUCUCAAG 3' — MRBY01000050 CRISPR N° 1 Spacer N° 52

3' GAGGAAATAAAAAGTACTATGCAAATAATATAGTATCATAATAATTATCAC 5' — ***Leuconostoc*** **phage phiLNTR2**
(position: 11665-11633)

13.

5' AGAGAAGCCCCACUGCGUAAUCUCCCCAGAAGGCGACGAUUUUUGUUUCCAA 3' — MRBY01000032 CRISPR N° 1 Spacer N° 2

3' AGGGGTAGGGGTTACATATTGGTGGGGTCTTCTGCTGGTATAAACGGCTGCG 5' — ***Prochlorococcus*** **phage P-TIM68**
(position: 178249-178214)

Supplementary Figure 4 continuation

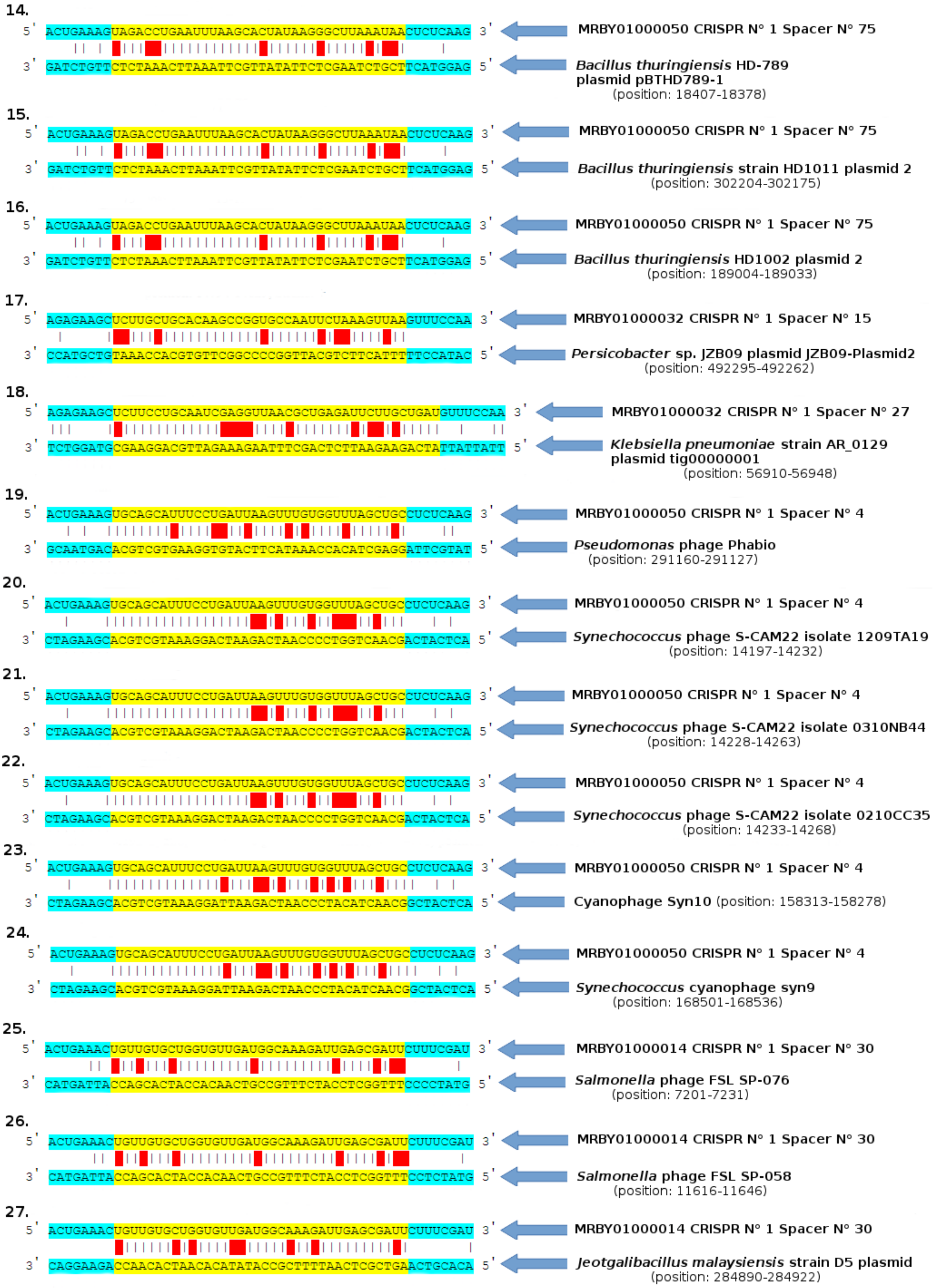

Supplementary Figure 4. Alignments of *Limnothrix* rosea IAM M-220 NIES-208 CRISPR spacers and targets (protospacers), predicted by CRISPRTarge

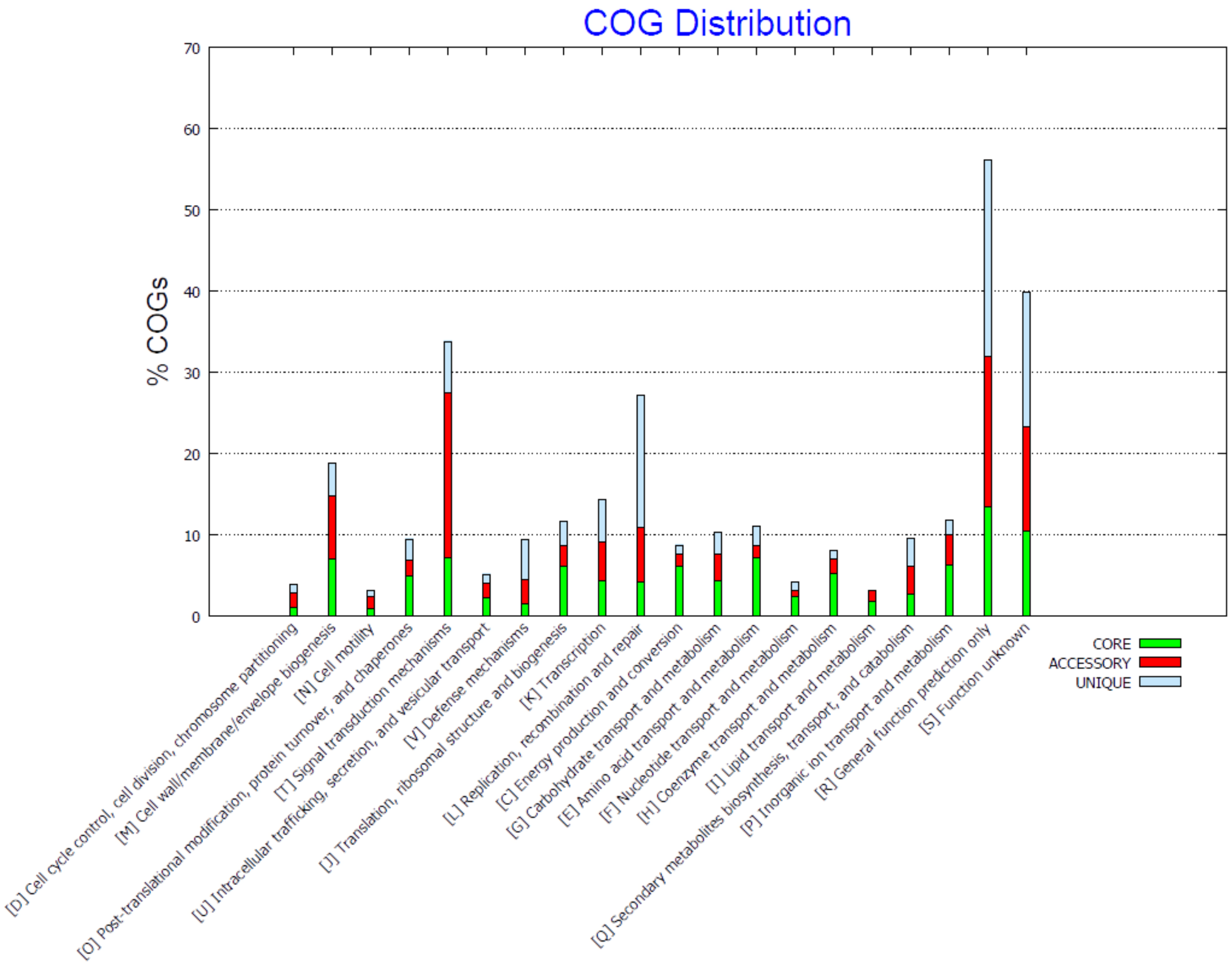

Supplementary Figure 5. COG distribution of core, accessory and unique genes present in three *Limnothrix* strains: CACIAM 69d, PR1529 and P13C2. Figure generated by Bacterial Pan Genome Analysis Tool (BPGA)

Worst Median Best

Statistics without reference	Newbler	SPAdes
# contigs	7181	9861
# contigs (>= 0 bp)	8433	9876
# contigs (>= 1000 bp)	5531	6679
# contigs (>= 5000 bp)	852	916
# contigs (>= 10000 bp)	450	501
# contigs (>= 25000 bp)	200	200
# contigs (>= 50000 bp)	77	65
Largest contig	241 377	251 784
Total length	27 701 667	30 579 508
Total length (>= 0 bp)	28 043 525	30 583 357
Total length (>= 1000 bp)	26 420 773	27 733 808
Total length (>= 5000 bp)	17 666 692	17 660 091
Total length (>= 10000 bp)	14 862 762	14 763 574
Total length (>= 25000 bp)	10 841 688	10 008 048
Total length (>= 50000 bp)	6 640 643	5 499 182
N50	13 349	8904
N75	2410	1718
L50	362	557
L75	1781	2899
GC (%)	61.11	61.29
**Mismatches**		
# N's	630	0
# N's per 100 kbp	2.27	0

Supplementary Table 1. CACIAM 69d assembly results for Newbler and SPAdes. Figure generated by QUAST. Only the best result for SPAdes (using k-mer 127) is shown.

Worst Median Best

Statistics without reference	Newbler MyCC	SPAdes MyCC
# contigs	122	99
# contigs (>= 0 bp)	122	99
# contigs (>= 1000 bp)	114	93
# contigs (>= 5000 bp)	90	83
# contigs (>= 10000 bp)	76	72
# contigs (>= 25000 bp)	55	56
# contigs (>= 50000 bp)	33	34
Largest contig	241 377	251 784
Total length	4 597 952	4 581 382
Total length (>= 0 bp)	4 597 952	4 581 382
Total length (>= 1000 bp)	4 592 401	4 576 189
Total length (>= 5000 bp)	4 545 711	4 554 247
Total length (>= 10000 bp)	4 446 663	4 473 906
Total length (>= 25000 bp)	4 108 984	4 227 867
Total length (>= 50000 bp)	3 303 677	3 442 792
N50	86 879	86 917
N75	45 306	50 181
L50	18	17
L75	37	34
GC (%)	55.21	55.21
**Mismatches**		
# N's	6	0
# N's per 100 kbp	0.13	0

Supplementary Table 2. MyCC results for the Cyanobacteria bin obtained from Newbler and SPAdes k-127 assemblies. Figure generated by QUAST.

Supplementary Table 3 – Number of protein sequences for representatives of core, accessory and unique orthologous clusters present in *Limnothrix* rosea *IAM M-220* and *Leptolyngbya* sp. PCC 7376 genomes.

Organism	No. of core genes	No. of accessory genes	No. of unique genes	No. of coding CDS
Limnothrix rosea IAM M-220	2,726	0	1,183	3,540
Leptolyngbya sp. PCC 7376	2,726	0	736	4,228

Supplementary Table 4 – Number of protein sequences for representatives of core, accessory and unique orthologous clusters present *Limnothrix* rosea *IAM M-220* and three *Leptolyngbya* genomes.

Organism	No. of core genes	No. of accessory genes	No. of unique genes	No. of coding CDS
Limnothrix rosea IAM M-220	1,002	1,815	648	3,540
Leptolyngbya sp. PCC 7376	1,002	1,805	1,104	4,228
Leptolyngbya sp. PCC 6406	1,002	988	2,323	4,905
Leptolyngbya sp.O 77	1,002	907	2,193	4,291

Supplementary Table 5 – Number of protein sequences for representatives of core, accessory and unique orthologous clusters present *Limnothrix* rosea *IAM M-220* and three *Oscillatoria* genomes.

Organism	No. of core genes	No. of accessory genes	No. of unique genes	No. of coding CDS
Limnothrix rosea IAM M-220	961	552	1,952	3,540
Oscillatoria sp. PCC 10802	961	1,500	2,452	6,536
Oscillatoria acuminata PCC 6304	961	1,697	2,704	5,879
Oscillatoria nigro-viridis PCC 7112	961	1,734	2,618	6,408

Supplementary Table 6– Number of protein sequences for representatives of core, accessory and unique orthologous clusters present *Limnothrix* rosea *IAM M-220* and two *Spirulina* genomes.

Organism	No. of core genes	No. of accessory genes	No. of unique genes	No. of coding CDS
Limnothrix rosea IAM M-220	1,313	269	1,886	3,540
Spirulina major PCC 6313	1,313	895	1,734	4,185
Spirulina subsalsa PCC 9445	1,313	890	1,837	4,353

Supplementary Table 7. List of genes related to assembly and export of extracellular polymeric substances in *Limnothrix* sp. CACIAM 69d, *Limnothrix* sp. P13C2, *Limnothrix* sp. PR1529 and *Limnothrix rosea* IAM M-220. For each gene, there is an indication of Pfam domain(s): annotation (locus tag). The whole genome Pfam profile was obtained using antiSMASH 4.0.

Gene	*Limnothrix* sp. CACIAM 69d	*Limnothrix* sp. P13C2	*Limnothrix* sp. PR1529	*Limnothrix rosea* IAM M-220 NIES-208
Wza, KpsD	**Poly_export:** polysaccharide export protein (BJG00_02265) polysaccharide transporter (BJG00_012635)	**Poly_export:** hypothetical protein (BCR12_12150, BCR12_11065)	**Poly_export:** hypothetical protein (AMR42_10600)	**Poly_export:** hypothetical protein (NIES208_15285)
Wzb	**LMWPc:** low molecular weight phosphotyrosine protein phosphatase (BJG00_003315) arsenate reductase, glutathione/glutaredoxin type (BJG00_011515)	**LMWPc:** protein tyrosine phosphatase (BCR12_09350) arsenate reductase, glutathione/glutaredoxin type (BCR12_09125)	**LMWPc:** ArsC family transcriptional regulator (AMR42_18100), protein tyrosine phosphatase (AMR42_08095)	**LMWPc:** protein tyrosine phosphatase (NIES208_08635) arsenate reductase, glutathione/glutaredoxin type (NIES208_12105)
Wzc, KpsE (Wzz)	**Wzz and GNVR:** hypothetical protein (BJG00_012630)	**Wzz and GNVR:** hypothetical protein (BCR12_11060)	No hits	**Wzz:** hypothetical protein (NIES208_15280)
Wzy (WaaL)	**Wzy_C and O-antigen_lig:** O-antigen ligase family protein (BJG00_017475, BJG00_002205) putative bicarbonate transporter, IctB family (BJG00_008780)	**Wzy_C and O-antigen_lig:** putative bicarbonate transporter, IctB family (BCR12_13670) hypothetical protein (BCR12_12080, BCR12_04350	**Wzy_C and O-antigen_lig:** polymerase (AMR42_15380), hypothetical protein (AMR42_04555)	**Wzy_C and O-antigen_lig:** O-antigen polymerase (NIES208_15775, NIES208_05280) putative bicarbonate transporter, IctB family (NIES208_17000) hypothetical protein (NIES208_00535, NIES208_01225, NIES208_07680)

Gene	*Limnothrix* sp. CACIAM 69d	*Limnothrix* sp. P13C2	*Limnothrix* sp. PR1529	*Limnothrix rosea* IAM M-220 NIES-208
Wzx	**Polysacc_synt, Polysacc_synt_C and Polysacc_synt_3:** teichoic acid transporter (BJG00_005715)<br>**Polysacc_synt_C and MatE + MVIN:** murein biosynthesis integral membrane protein MurJ (BJG00_006465)	**Polysacc_synt, Polysacc_synt_C and Polysacc_synt_3:** hypothetical protein (BCR12_07660)<br>**Polysacc_synt_C and MatE + MVIN:** murein biosynthesis integral membrane protein MurJ (BCR12_14465)	**Polysacc_synt, Polysacc_synt_C and Polysacc_synt_3:** hypothetical protein (AMR42_14170)<br>**Polysacc_synt_C and MatE + MVIN:** hypothetical protein (AMR42_04695)	**Polysacc_synt, Polysacc_synt_C and Polysacc_synt_3:** polysaccharide biosynthesis protein (NIES208_18195)<br>**Polysacc_synt_C and MatE + MVIN:** murein biosynthesis integral membrane protein MurJ (NIES208_04875)<br>MATE family efflux transporter (NIES208_14940)<br>**Polysacc_synt, Polysacc_synt_C, Polysacc_synt_3, MatE and MVIN**: O-unit flippase (NIES208_17565)<br>**Polysacc_synt, Polysacc_synt_C, Polysacc_synt_3 and MatE:** flippase (NIES208_00530)<br>**Polysacc_synt_C and MatE:** MATE family efflux transporter (NIES208_04535)
KpsM (Wzm)	**ABC2_membrane and ABC2_membrane_3:** ABC transporter ATP-binding protein (BJG00_017165, BJG00_017750)	**ABC2_membrane and ABC2_membrane_3:** ABC transporter permease (BCR12_14910, BCR12_07015)<br>ABC transporter substrate-binding protein (BCR12_11995)<br>Transporter (BCR12_15220)	**ABC2_membrane and ABC2_membrane_3:** ABC transporter permease (AMR42_04595, AMR42_04385), ABC transporter substrate-binding protein (AMR42_14810)	**ABC2_membrane and ABC2_membrane_3:** multidrug ABC transporter permease (NIES208_06540)<br>ABC transporter permease (NIES208_07575), NIES208_08175, NIES208_14570)<br>ABC transporter substrate-binding

Gene	*Limnothrix* sp. CACIAM 69d	*Limnothrix* sp. P13C2	*Limnothrix* sp. PR1529	*Limnothrix rosea* IAM M-220 NIES-208
				protein (NIES208_16725) transporter (NIES208_17485)
KpsT (Wzt)	**ABC_tran and Wzt_C:** nitrate ABC transporter ATP-binding protein (BJG00_000130)	**ABC_tran and Wzt_C:** nitrate ABC transporter ATP-binding protein (BCR12_00030)	**ABC_tran and Wzt_C:** ABC transporter ATP-binding protein(AMR42_18320)	**ABC_tran and Wzt_C:** ABC transporter ATP-binding protein (NIES208_03825)
KpsU	**CTP_transf_3:** 2-C-methyl-D-erythritol 4-phosphate cytidylyltransferase (BJG00_001410)	**CTP_transf_3:** 2-C-methyl-D-erythritol 4-phosphate cytidylyltransferase (BCR12_10010)	**CTP_transf_3:** 2-C-methyl-D-erythritol 4-phosphate cytidylyltransferase (AMR42_10190)	No hits
Alg8, BcsA	**Glyco_tranf_2_3:** glycosyl transferase family 2 (BJG00_010415, BJG00_016725, BJG00_017305, BJG00_017595, BJG00_017700, BJG00_018595, BJG00_001030, BJG00_003325) Glycosyltransferase (BJG00_010625, BJG00_010630, BJG00_010755, BJG00_012895, BJG00_012900, BJG00_013110, BJG00_013365, BJG00_015675, BJG00_015900, BJG00_017490, BJG00_018720, BJG00_018875, BJG00_000120, BJG00_001560, BJG00_001765, BJG00_003915, BJG00_003935, BJG00_006395, BJG00_006485, BJG00_007100, BJG00_009780)	**Glyco_tranf_2_3:** glycosyl transferase family 2 (BCR12_08915, BCR12_04365, BCR12_09860) Glycosyltransferase (BCR12_16595, BCR12_16600, BCR12_06280, BCR12_12705, BCR12_12710, BCR12_08780, BCR12_06500, BCR12_02990, BCR12_09665, BCR12_04455) hypothetical protein (BCR12_00040, BCR12_08395, BCR12_11000, BCR12_11565, BCR12_14860, BCR12_09340, BCR12_08130, BCR12_11790, BCR12_15085, BCR12_08210, BCR12_07290, BCR12_05150, BCR12_14400, BCR12_07750, BCR12_14480, BCR12_10695)	**Glyco_tranf_2_3:** hypothetical protein (AMR42_02875, AMR42_02955, AMR42_03190, AMR42_04175, AMR42_05805, AMR42_06095, AMR42_11055, AMR42_12135, AMR42_13035, AMR42_16840, AMR42_18310) glycosyl transferase family 2 (AMR42_03345, AMR42_10040, AMR42_11940) glycosyl transferase (AMR42_03980, AMR42_03985, AMR42_05620, AMR42_08410, AMR42_11595, AMR42_12325, AMR42_12330, AMR42_13625, AMR42_17265, AMR42_17485) sulfonate ABC transporter permease (AMR42_12205)	**Glyco_tranf_2_3:** hypothetical protein (NIES208_00005, NIES208_09865, NIES208_13230) glycosyl transferase family 2 (NIES208_00025, NIES208_06300, NIES208_10020, NIES208_17215) glycosyl transferase (NIES208_00015, NIES208_00700, NIES208_03210, NIES208_04665, NIES208_05125, NIES208_07560, NIES208_14450)

Gene	*Limnothrix* sp. CACIAM 69d	*Limnothrix* sp. P13C2	*Limnothrix* sp. PR1529	*Limnothrix rosea* IAM M-220 NIES-208
	tetratricopeptide repeat protein (BJG00_004295) DUF2064 domain-containing protein (BJG00_005820)	sulfonate ABC transporter permease (BCR12_12830)		
AlgG	**NosD:** DUF1565 domain-containing protein (BJG00_006605, BJG00_011735, BJG00_014985)	**NosD:** nitrous oxidase accessory protein (BCR12_16285) hypothetical protein (BCR12_14610, BCR12_01710)	**NosD:** nitrous oxidase accessory protein (AMR42_19300), hypothetical protein (AMR42_07275)	**NosD:** hypothetical protein (NIES208_10725, NIES208_00115, NIES208_01685)
AlgI	**MBOAT:** MBOAT family protein (BJG00_003710)	**MBOAT:** alginate O-acetyltransferase (BCR12_01905)	**MBOAT:** alginate O-acetyltransferase (AMR42_07470)	**MBOAT:** membrane-bound O-acyltransferase family protein (NIES208_16120)
ExoD	No hits	No hits	No hits	**ExoD:** hypothetical protein (NIES208_09365)
